# Biclustering data analysis: a comprehensive survey

**DOI:** 10.1093/bib/bbae342

**Published:** 2024-07-15

**Authors:** Eduardo N Castanho, Helena Aidos, Sara C Madeira

**Affiliations:** LASIGE, Faculdade de Ciências, Universidade de Lisboa, Campo Grande 16, P-1749-016 Lisbon, Portugal; LASIGE, Faculdade de Ciências, Universidade de Lisboa, Campo Grande 16, P-1749-016 Lisbon, Portugal; LASIGE, Faculdade de Ciências, Universidade de Lisboa, Campo Grande 16, P-1749-016 Lisbon, Portugal

**Keywords:** biclustering, biclustering taxonomy, biclustering algorithms, biclustering evaluation, heterogeneous biclustering, biclustering-based classification

## Abstract

Biclustering, the simultaneous clustering of rows and columns of a data matrix, has proved its effectiveness in bioinformatics due to its capacity to produce local instead of global models, evolving from a key technique used in gene expression data analysis into one of the most used approaches for pattern discovery and identification of biological modules, used in both descriptive and predictive learning tasks. This survey presents a comprehensive overview of biclustering. It proposes an updated taxonomy for its fundamental components (bicluster, biclustering solution, biclustering algorithms, and evaluation measures) and applications. We unify scattered concepts in the literature with new definitions to accommodate the diversity of data types (such as tabular, network, and time series data) and the specificities of biological and biomedical data domains. We further propose a pipeline for biclustering data analysis and discuss practical aspects of incorporating biclustering in real-world applications. We highlight prominent application domains, particularly in bioinformatics, and identify typical biclusters to illustrate the analysis output. Moreover, we discuss important aspects to consider when choosing, applying, and evaluating a biclustering algorithm. We also relate biclustering with other data mining tasks (clustering, pattern mining, classification, triclustering, N-way clustering, and graph mining). Thus, it provides theoretical and practical guidance on biclustering data analysis, demonstrating its potential to uncover actionable insights from complex datasets.

## Introduction

**Figure 1:**
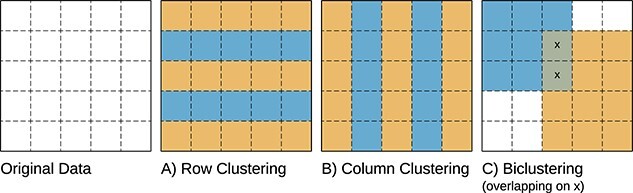
Differences between **A**) Row Clustering, **B**) Column Clustering, and **C**) Biclustering; while (hard) clustering methods search for disjoint groups of rows or columns in the data matrix (global model), biclustering discovers sub-matrices satisfying homogeneity and statistical significance criteria (local model); the orange and blue colors show two row clusters (A), two column clusters (B), and two overlapped biclusters (C).

*Biclustering*, also referred to as *co-clustering* or *two-way clustering*, is a machine learning technique that simultaneously groups rows (observations) and columns (attributes) of a data matrix. By generalizing traditional clustering methods, it uncovers complex relationships between observations [1]. Figure 1 illustrates the differences between clustering and biclustering.

Biclustering has three conceptual benefits when compared with traditional clustering algorithms (such as K-means and hierarchical clustering): **first**, biclustering acknowledges that similarity between observations (rows) exists exclusively in a subset of the attributes (columns), contrasting with clustering that considers all attributes when computing the similarity between observations. This property makes biclustering suited for the analysis of biological data characterized by local patterns, in particular gene expression data, discovering transcriptional modules composed of a subset of genes (rows) correlated in subsets of samples (columns) [2]. **Second**, biclustering allows for overlapping, meaning that both an observation and an attribute can simultaneously belong to several groups (hard clustering forces observations to a single group), reflecting the simultaneous participation of genes in multiple biological processes [3–5]. **Third**, it is more flexible in detecting complex relationships between observations, capturing hidden structures and patterns that are not evident when analyzing data using global models [6, 7]. The versatility of biclustering is evidenced by its capacity to detect composite contributions from simultaneous biological processes with overlapping signals, unveil diverse biological patterns, and utilize data-driven methodologies tailored to the nuances of each specific research problem [5, 8–11].

**Figure 2:**
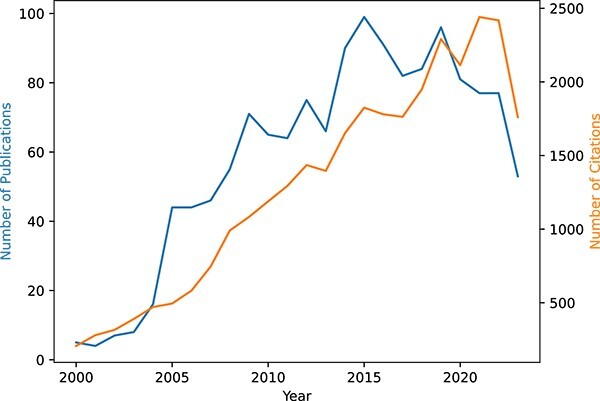
Evolution of biclustering as a scientific field since the publication of Cheng and Church’s algorithm, measured by the number of publications and citations, considering results the Web of Science using *biclustering* and *coclustering* as keywords since 2000.

Biclustering was first introduced by Hartigan in 1972 [12], became popular in biological and biomedical domains with the development of Cheng and Church’s algorithm applied for gene expression data in 2000 [2]. Today, biclustering is state of the art for analyzing correlations between a subset of genes and subsets of experimental conditions, identifying modules in biological networks, stratifying patients by phenotype, and analyzing gene–drug associations [7]. Beyond bioinformatics, several studies have expanded the usefulness of biclustering in domains such as text mining, recommendation systems, and climate science [1, 13–17]. Figure 2 shows the growth of biclustering as a scientific field since 2000. Different studies reflect this growth, focusing on specific aspects of biclustering data analysis:

- **Algorithmic Studies**: analyze the algorithmic characteristics of a selected number of biclustering algorithms [4, 5, 18–22];
- **Comparative Studies**: quantitatively compare the performance of biclustering algorithms [3, 20, 23–27];
- **Measure Studies**: analyze evaluation metrics used by the algorithms and comparative studies [28–32];
- **Application Studies**: examine the use of biclustering in specific applicational domains [7, 14, 33];
- **Software Studies**: present software associated with biclustering analysis [8, 34–37].

Instead of focusing on a specific aspect of biclustering, this survey provides a comprehensive overview of the biclustering task and its main components (bicluster, biclustering solution, biclustering algorithms, evaluation measures) and applications. The closest survey was published in 2004 by Madeira and Oliveira [1]. Our work expands beyond the 2004 survey by incorporating insights and conclusions from previous studies [5, 18, 20, 21, 31, 38] regarding the application of biclustering while following the same computational view in the methodological sections, updated to reflect the advances in biclustering concepts and algorithms, and to relate them to the application domains. We aim to provide theoretical and practical guidance about biclustering data analysis, showing its potential to unravel actionable knowledge from data and highlight prominent application domains.

**Figure 3:**
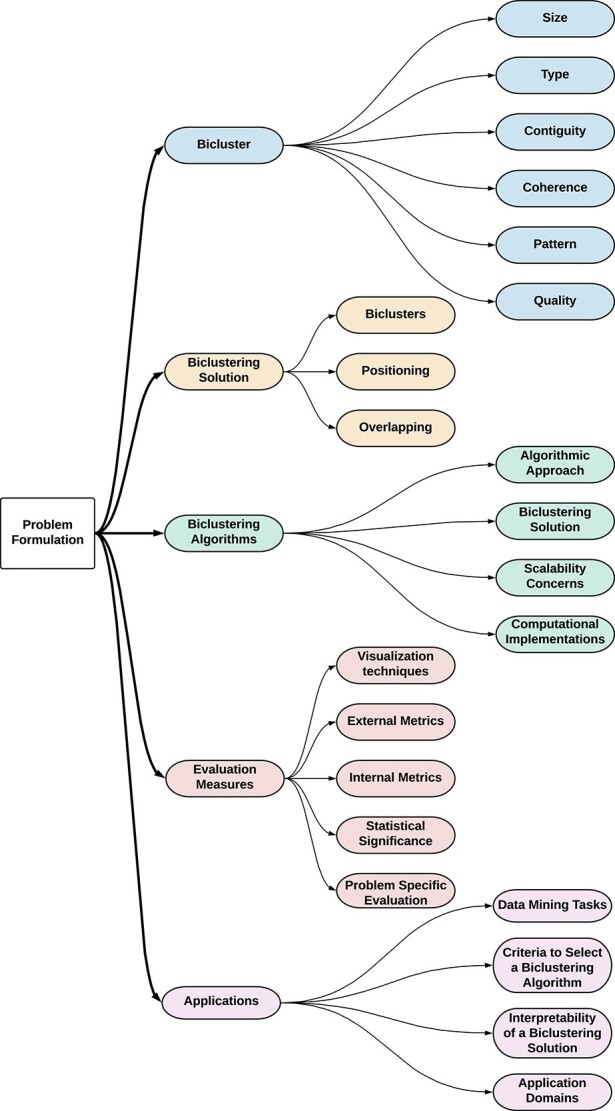
Taxonomy of biclustering as proposed by our survey.

Our manuscript has two parts: *first*, it discusses theoretical aspects of biclustering using the taxonomy shown in Figure 3, incorporating discussions from previous studies with new concepts, targeting a unified taxonomy of biclustering. These theoretical concepts accommodate both the expansion of biclustering to new data types and the specificities of applicational domains. The *second* part of this study targets the use of biclustering in applicational studies, particularly in biological and biomedical data. We first relate conceptually biclustering with other data mining tasks, then discuss practical guidance for choosing a biclustering algorithm and analyze the algorithm’s solution. Finally, we identify relevant application areas given the biclustering literature.

Accordingly, this survey follows the taxonomy sections: we discuss a pipeline for biclustering data analysis and formalize the task in **Problem Formulation**. Sections **Bicluster**, **Biclustering Solution**, **Biclustering Algorithm**, and **Evaluation Measures** provide the theoretical concepts and list popular biclustering algorithms and measures. In **Applications**, we focus on the relationship between biclustering and other data mining tasks, provide practical aspects of biclustering data analysis (such as selecting an adequate algorithm and analyzing the solution), and the use of biclustering in biological and biomedical applications. **Conclusion** provides final remarks and discusses challenges for biclustering data analysis.

## Problem formulation

Biclustering is an unsupervised machine learning task that simultaneously groups rows (observations) and columns (attributes) of a data matrix. Being an unsupervised approach, it does not rely on ground truth or use labeled observations. Biclustering algorithms can be applied to analyze a large diversity of data, such as:

- *Network data:* modeling networks of biological entities, such as protein–protein interactions or gene–drug associations [9, 39];
- *Time Series:* sequences of data points collected at successive points, such as studying different attributes along time, brain BOLD signal or gene expression in response to stimuli [26, 40];
- *Time Points:* identifying the time at which an event occurs, such as the time when a biopsy was performed [41];
- *Coordinates:* latitude/longitude pairs identifying an exact location [42];
- *Locations:* identifying a place or an area, such as the place of residence of a patient [43];
- *Text:* associated with the analysis of a text, such as research papers or medical records [44];
- *Sequences:* referring to ordered sequences such as DNA sequences to find motifs [45, 46];
- *Figures:* relating to figures such as photografies or brain MRI scans [47, 48].

**Figure 4:**
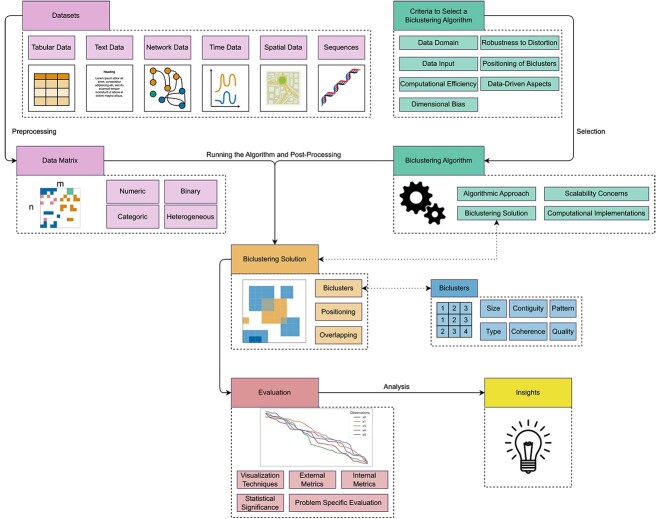
Pipeline for biclustering data analysis; first, the datasets must be converted into data matrices, as biclustering algorithms require this data format; selecting an adequate biclustering algorithm is key to guarantee that it is adequate for the data under analysis and that the results (biclustering solution) meet the problem-specific questions; finally, the solution must be analyzed using quantitative and/or qualitative techniques to evaluate its quality and extract meaningful insights from the data; each topic in this pipeline is discussed in the proposed biclustering taxonomy in a dedicated section.

Any biclustering data analysis pipeline, illustrated in Figure 4, begins by transforming the original data into data matrices. A **Data Matrix**$A$ is defined by $N$**observations** (rows), $X = \{x_{1},..., x_{N}\}$, and $M$**attributes** (columns), $Y = \{y_{1},...,y_{M}\}$. An **element** of the matrix $a_{ij}$ is a value relation defined for each observation $x_{i}$ and attribute $y_{j}$.

**Figure 5:**
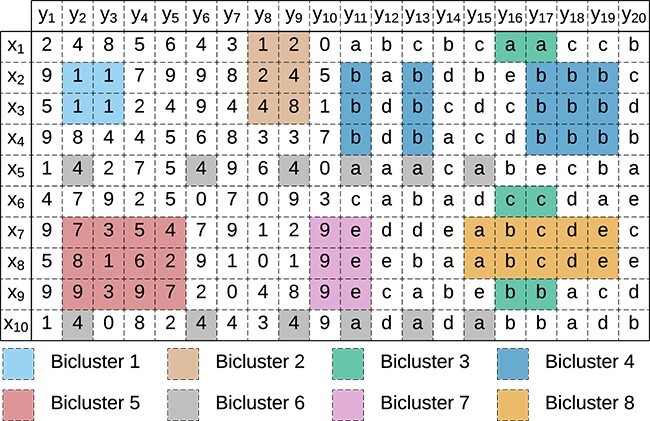
Example of a mixed-attribute data matrix with eight nonoverlapping biclusters.

Biclustering analysis aims to extract and analyze biclusters from these data matrices. A **Bicluster**$B=(I,J)$ is a subset of rows $I \subseteq X$ and columns $J \subseteq Y$ of the original matrix, $n$ and $m$ represent the number of rows and columns of the bicluster, and $b_{ij}$ denotes the element of $B$ corresponding to row $x_{i}$ and column $y_{j}$. Figure 5 illustrates a set of biclusters in a mixed-attribute data matrix.

A **Biclustering Algorithm** aims to discover a set of biclusters such that each bicluster satisfies criteria of homogeneity and statistical significance. The output of the algorithm is a **Biclustering Solution**, or Biclustering in short. A biclustering (solution) is thus the set of $q$ biclusters $\mathcal{B} = \{B_{1},\cdots , B_{q}\}$ discovered by the biclustering algorithm. The properties of the found biclustering depend on the algorithm. Table 1 summarizes the mathematical notation used in this survey.

**Table 1:** Mathematical notation followed in this survey

Symbol	Explanation
$A$	Data Matrix of the dataset
$X$	Rows of the data matrix
$Y$	Columns of the data matrix
$a_{ij}$	Element of the data matrix
$N$	Number of rows of the data matrix
$M$	Number of columns of the data matrix
$x_{i}$	Row index
$y_{j}$	Column index
$B$	Bicluster
$I$	Rows of the bicluster
$J$	Columns of the bicluster
$b_{ij}$	Element of a bicluster
$n$	Number of rows of the bicluster
$m$	Number of columns of the bicluster
$P$	Pattern of a bicluster
$\mathcal{B}$	Biclustering
$q$	Number of biclusters in a biclustering $\mathcal{B}$
$\dot{\mathcal{B}}$	Reference Bicluster
$S(\mathcal{B},\dot{\mathcal{B}})$	External Measure

## Bicluster

A bicluster is the atomic element of biclustering and consists of a submatrix of the original data matrix. A bicluster is defined by its **Size**, **Type**, **Contiguity**, **Coherence**, **Pattern**, and **Quality**, discussed on the following subsections. Several definitions from this section follow Madeira and Oliveira’s survey [1].

### Size

A bicluster is defined by its size, i.e. **number of rows**$n$, and **columns**$m$, and **area**$n\times m$. If $I = X$ or $J = Y$, the bicluster is a *Column* or *Row Cluster*, recovering definitions from traditional clustering.

A bicluster with less than two rows or columns is known as a *Degenerate* or *Trivial Bicluster*. While some algorithms output these biclusters, they have little interest since they are hardly found in real applications and are thus often removed from the analysis in a post-processing step [28].

### Type

The attributes of the data matrix define the type of a bicluster. **Homogeneous biclusters** consist exclusively of traditional attributes with the same data type:

- **Numeric Biclusters:** biclusters with numeric attributes. It further expands as *Integer*, referring to non-decimal values (such as the number of visits to a hospital), *Ratio*, referring fractions (such as saturation of oxygen in the blood), and *Real-valued*, with decimal-valued attributes (such as blood pressure);
- **Categorical Biclusters:** biclusters where attributes fall in discrete categories. It further expands as *Nominal*, where attributes have no natural ordering (such as blood type), or *Ordinal*, where there is an ordering between the categories (such as the stage of a disease);
- **Binary Biclusters:** biclusters with binary values. It further expands as *Symmetric*, where the two values are equally important (such as biological sex), or *Asymmetric*, where one value is more important than the other (such as having a gene mutation).

**Heterogeneous Biclusters** consider data beyond homogeneous types, incorporating attributes beyond traditional tabular data, such as the analysis of networks and spatial-temporal data. A particular case of heterogeneous biclusters is mixed biclusters, which incorporate attributes of different types [49]. Table 2 organizes biclustering algorithms and studies for each bicluster type.

**Table 2:** Biclustering algorithms and application domains organized by the type of the bicluster; biclustering was originally a tool to mine and analyze biclusters obtained from numerical matrices (particularly gene expression data matrices) and evolved to analyze datasets with diverse data characteristics and diverse applicational domains

Homogeneity	Type	Application domains	Example Algorithms
Homogeneous	Numeric	Gene Expression [7] Metabolical [50] Pharmacology [51] Agriculture [52, 53] Plant Biology [54]	ISA [55] SAMBA [56] QUBIC [57] XMotifs [58] Fabia [59]
	Categorical	Used with numerical data in mixed datasets.	BicPAM [8] BicSPAM [60] Kpax3 [46]
	Binary	Bibliometric [61, 62] Gene Expression [63]	Bimax [64] BiBit [65]
Heterogeneous	Networks	Biological Networks [66, 67] Mobility Networks [68]	BicNet [9] Bimax [64] BiBit [65]
	Time Series	Gene Expression [36] Phisiological [26] Resources Consuption [69] Clinical [70, 71] Urban Mobility [72]	CCC-Biclustering [11] e-CCC-Biclustering [73] LateBiclustering [74]
	Mixed	Clinical [75–78]	HBC [75] BicPAM [8]
	Time Points	Clinical [41]	HBC-t [41]
	Spatial Locations	Phisiological [26, 79] Climate [15, 17, 80, 81] Social Sciences [82, 83] Epidemics [84, 85]	Cheng and Church [2] ISA [55]

### Contiguity

**Figure 6:**
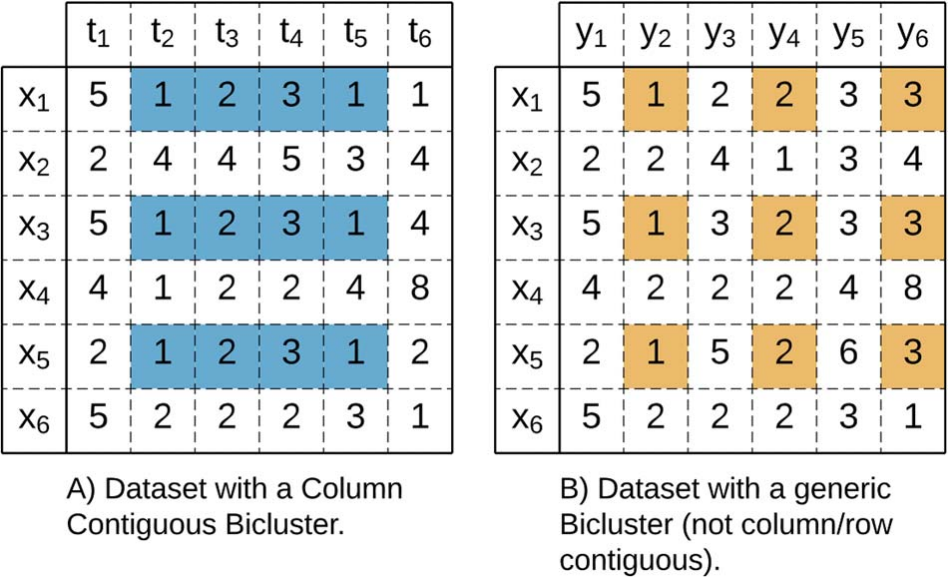
When the ordering between the columns in the data matrix is relevant, **A**) (Column) Contiguous biclusters have interpretability advantages compared with **B**) generic biclusters.

A contiguous bicluster (**C-Bicluster**) has an ordering considering either its rows or columns (Figure 6 illustrates a column contiguous bicluster). This is relevant for domains with an assumed ordering, such as time series datasets (where columns identify ordered time points). Examples of contiguous biclusters are seen in diverse scientific domains such as gene expression data, where the temporal change in expression patterns is used to monitor complex biological processes such as disease progression and drug responses [86], fMRI data, where biclusters measure an activity pattern in several regions [26, 87], and resources utilization, measured over time (usually expressed in the columns) [69, 88]. In this context, the contiguous column bicluster (CC-Bicluster) was defined [11] and expanded [73, 74], with interpretive advantages.

### Coherence

**Figure 7:**
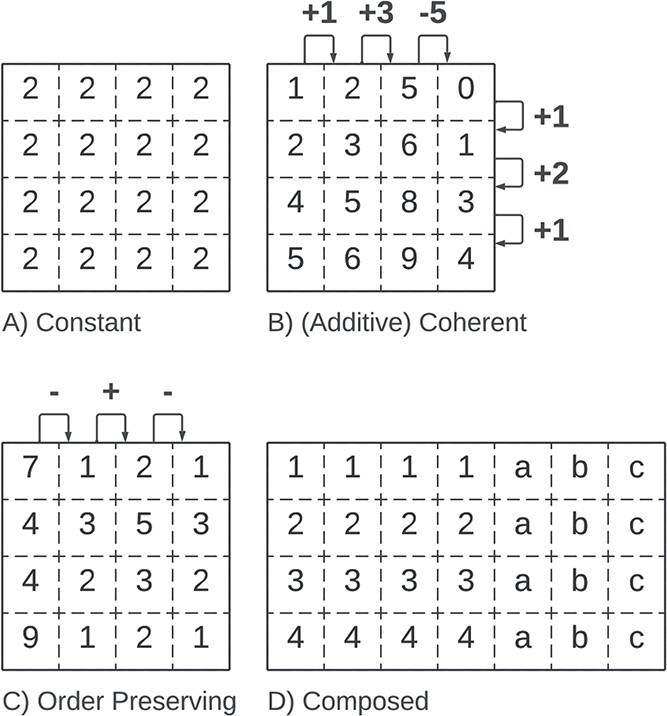
The coherence assumption of a bicluster explains the correlation between values; possible coherency assumptions are **A**) Constant (when the rows or columns have equal values), **B**) Coherent (a mathematical model explains the variability inside the bicluster), **C**) Order Preserving (where the values inside the bicluster follow a common trend), and **D**) Composed (the bicluster joins multiple biclusters with different types/coherences).

The coherence of a bicluster explains the assumed correlations between the bicluster elements in the absence of noise. In this survey, we consider four coherence assumptions, illustrated in Figure 7: **Constant**, **Coherent**, **Order-Preserving**, and **Composed**. Table 3 illustrates the relevance of each coherence with examples of biologically mined biclusters. In the remaining section, we explain each coherency assumption and corresponding sub-coherences.

**Table 3:** Biological examples of biclusters with each coherency assumption; biclustering algorithms detect structures with flexible definitions of similarity between observations in a data matrix, thus identifying a large diversity of biological phenomena

Coherency	Biological examples
Constant	Co-expressed genes with a regulatory pattern across a subset of conditions [89, 90]; Dense regions in a binary biological network [65, 91, 92].
Coherent	Biological modules with additive and multiplicative factors measuring the responsiveness of biological entities [89, 93]. Dense regions in a weighted biological network [9].
Order Preserving	Genes with coherent evolutions in their expression levels [11, 60, 94].
Composed	Symmetric modules reflecting activation and repression mechanisms within transcriptomic, proteomic, or metabolic data [60].

#### Constant

**Figure 8:**
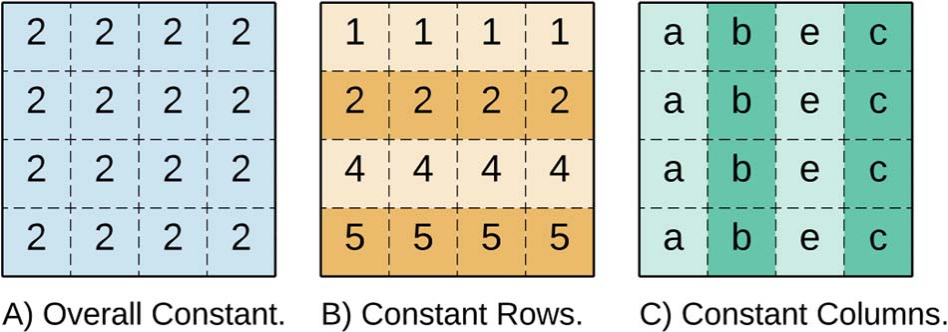
Constant Biclusters: **A**) Overall ($\mu = 2$), **B**) Constant Rows ($\alpha _{i} = [1,2,4,5]$), and **C**) Constant Columns ($\beta _{j} = [a,b,e,c]$).

A perfect *overall constant* bicluster, illustrated in Figure 8, is a sub-matrix where the elements in the bicluster equal a constant $\mu $, i.e. $b_{ij} = \mu $. Beyond the overall constant biclusters, a bicluster with *constant rows* has all values equal per row, i.e. $b_{ij} = \alpha _{i}$. Similarly, biclusters with *constant columns* are defined by $b_{ij} = \beta _{j}$.

#### Coherent

**Figure 9:**
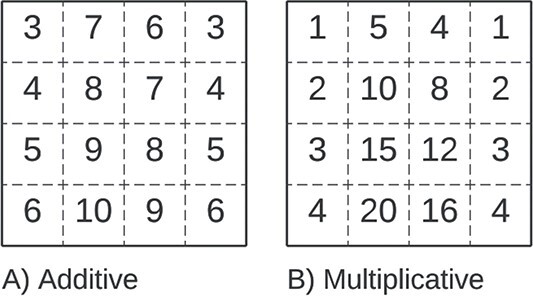
Coherent Biclusters are explained by a mathematical model, either considering an **A**) Additive relationship between rows and columns, or a **B**) Multiplicative relationship; both biclusters in the figure were generated with $\mu =1$, $\alpha _{i} = [1,2,3,4]$, and $\beta _{j} = [1,5,4,1]$.

A *coherent bicluster*, illustrated in Figure 9, defines a coherency exclusive to numerical biclusters and assumes simultaneous corrections in rows and columns. An *additive coherent bicluster*, also known as a *shifting bicluster*, is defined as $b_{ij} = \mu + \beta _{j} + \alpha _{i}$. A *multiplicative coherent bicluster*, also known as a *scaling bicluster*, is defined as $b_{ij} = \mu \times \beta _{j} \times \alpha _{i}$.

#### Order preserving

**Figure 10:**
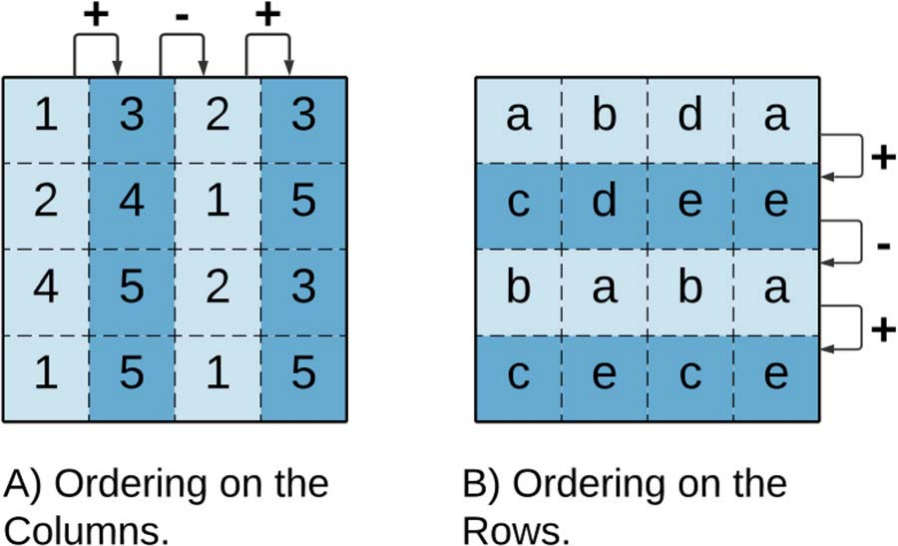
Order Preserving Biclusters represent general trends in the data (such as *up-down-up*) rather than explaining well-defined values; the order preserving coherency is defined on either **A**) columns or **B**) rows (assuming $a<b<c<d<e$).

*Order preserving* is a coherency valid for both numeric and ordinal categorical biclusters [94, 95]. In order preserving, illustrated in Figure 10, the biclusters’ rows (or columns) can be permuted such that the values increase monotonically. This coherency describes general trends in data, regardless of the exact values.

#### Composed

**Figure 11:**
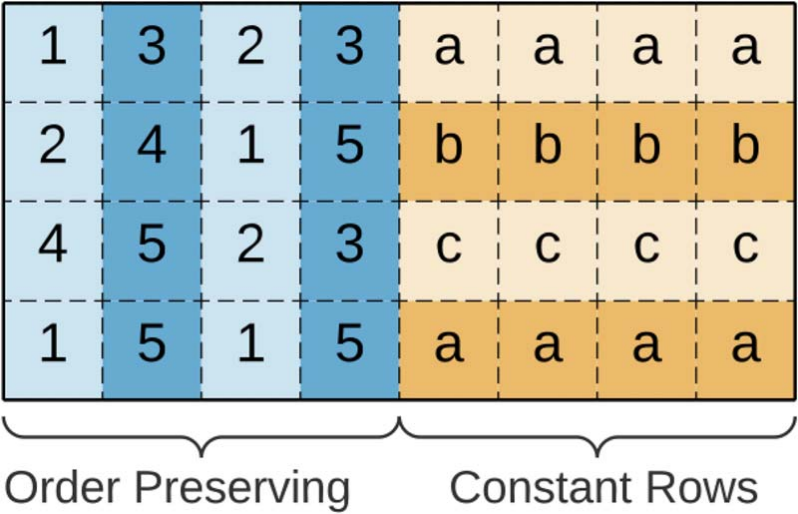
Composed biclusters consist of biclusters with more than one coherence; in this example, this bicluster combines two sub-biclusters: one order preserving on columns bicluster in the numeric attributes and one constant rows bicluster in the categorical attributes.

*Composed biclusters*, illustrated in Figure 11, have splitted coherence. These biclusters’ sub-matrices can be split into several sub-biclusters, each having its coherence. An example of composed biclusters are biclusters with an order preserving on columns coherency in the numerical attributes and constant rows in categorical attributes.

### Pattern

**Figure 12:**
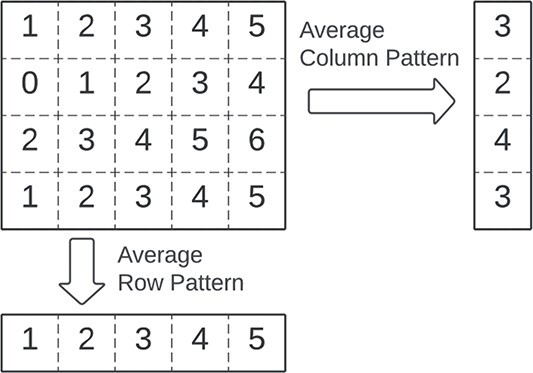
A bicluster has a pattern over its rows and its columns; the pattern of a bicluster corresponds simplifying the information inside it for easier analysis; it can be seen as the bicluster representant, as happens, for instance, with centroids in clustering.

**Figure 13:**
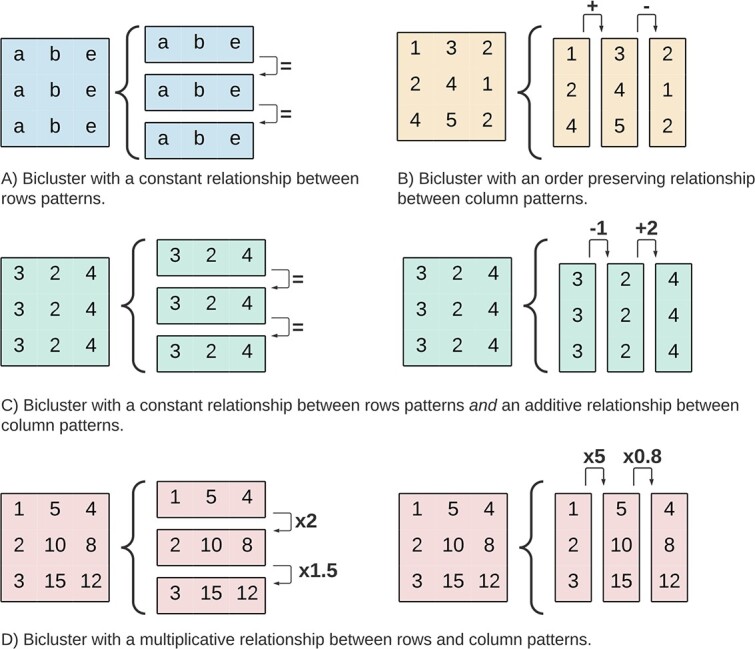
A bicluster can be described based on the relationship between row or column patterns; a pattern-centric description of biclustering allows for easy identification of coherences and noises in the biclusters.

The **pattern** of a bicluster is used to simplify and describe the bicluster (illustrated in Figure 12) [8, 20, 96]. Since a bicluster is two-dimensional, a pattern can be defined on the *rows* or over the *columns*. As illustrated in Figure 13, these patterns can have a constant, additive, multiplicative, or order preserving relationship. A pattern-based description of biclustering allows for improved computational searches and identification of complex relationships.

### Quality

Bicluster **quality** explores how the values fall out of the assumed pattern. This can be due to the presence of **missing values** inside the bicluster or due to **noise** [97, 98].

## Biclustering solution

A biclustering solution, also known as biclustering, is the set of $q$ biclusters $\mathcal{B} = \{B_{1},\cdots , B_{q}\}$ discovered by a biclustering algorithm. A biclustering is defined by its **biclusters**, its **positioning**, and **overlapping** between the elements of the biclusters.

### Biclusters

A biclustering solution is described by the **properties** of its biclusters. A biclustering solution has *defined type* if all biclusters have equal type:

- A **numeric biclustering** consists exclusively of numeric biclusters;
- A **categorical biclustering** consists exclusively of categorical biclusters;
- An **homogeneous biclustering** consists of either numeric or categoric biclusters.

In contrast to homogeneous biclustering solutions, if the solution includes at least one heterogeneous bicluster, then the solution is a **hetereogeneous biclustering**. The biclustering solution has **defined coherency** if all biclusters are assumed to have the same coherency.

### Positioning

**Figure 14:**
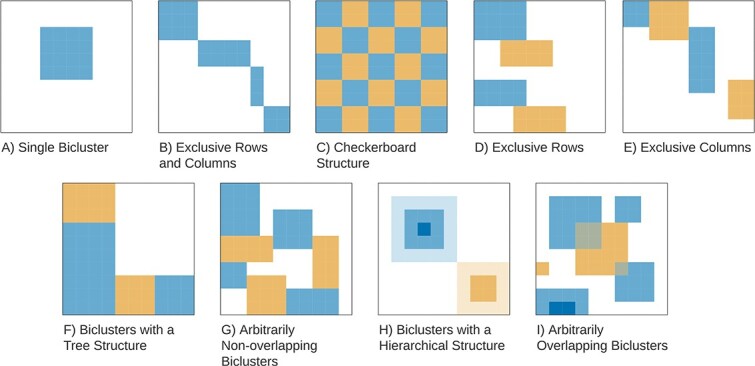
Biclustering structures; the structure of a biclustering solution is a consequence of both the algorithmic search of the biclusters and the intrinsic properties of the data matrix; none of these structures forces a full coverage or every observation/attribute to belong to a bicluster; figure adapted from Madeira and Oliveira [1].

In this section, we describe the biclustering solution based on the relative positioning of its biclusters, considering both its coverage and structure. Regarding **Coverage**, a biclustering solution has *Full Coverage* if every element of the matrix belongs to at least one bicluster, having *Partial Coverage* otherwise [21, 22]. The **Structure** of a biclustering refers to the relative position between the biclusters. Figure 14 illustrates the most popular taxonomy, as defined by Madeira and Oliveira [1].

The structure of a biclustering solution is a relevant aspect when selecting the biclustering algorithm, as the restrictions it imposes in the biclustering solution guide the analysis. The simplest structure is the case of a biclustering solution consisting of a *single bicluster* (Figure 14 A)). Any algorithm that allows control over the number of biclusters can be used to obtain a single bicluster. An issue with this approach is that there are no guarantees that this obtained bicluster is “the best” the algorithm can discover (as sometimes it is only the first mined). In alternative, a biclustering solution of one bicluster can be obtained by filtering any biclustering solution according to a criterion.

Beyond the single bicluster, *Exclusive Rows and Columns* (Figure 14 B)) partitions the data matrix. This partitioning strategy allows for easy interpretability of the biclusters. However, it ignores the possibility that observations can belong to multiple processes and, thus, multiple biclusters. A generalization of this structure is the *Checkerboard Structure* (Figure 14 C)) that divides the data matrix such as each observation and attribute belongs to multiple biclusters, not necessarily forcing each row and column to belong to a bicluster. *Exclusive Rows* and *Exclusive Columns* (Figure 14 D) and E)) are structures close to traditional clustering algorithms, as D) can be seen as row clustering with an automatic selection of relevant attributes (equivalent for E)). *Biclusters with a Tree Structure* and *Arbitrarily Nonoverlapping Biclusters*Figure 14 D) and E) correspond to cases where there is no overlapping between biclusters, but the biclusters have a less rigid placement in the data matrix.

Discovering nonoverlapping structures has two advantages for biclustering analysis: first, the number of generated biclusters is typically smaller. Second, since the biclusters’ elements do not overlap, biclusters can be analyzed separately (similarly to traditional clustering analysis), facilitating interpretation. However, biclustering solutions with overlapping between their elements reflect broader patterns inside the data matrix and relevant problem-specific interactions.

*Biclusters with a Hierarchical Structure* (Figure 14 H)) consider disjoint sets of biclusters. For each set, a bicluster must include other. *Arbitrarily positioned overlapping biclusters* (Figure 14 I)) refers to the most general case, where there is no particular structure between the biclusters. In this structure, the overlapping between elements implies that it is harder to extract valuable knowledge from the biclustering. However, biclusters that search for this structure tend to achieve better results in comparative studies [3, 7, 20, 26, 69].

### Overlapping

**Figure 15:**
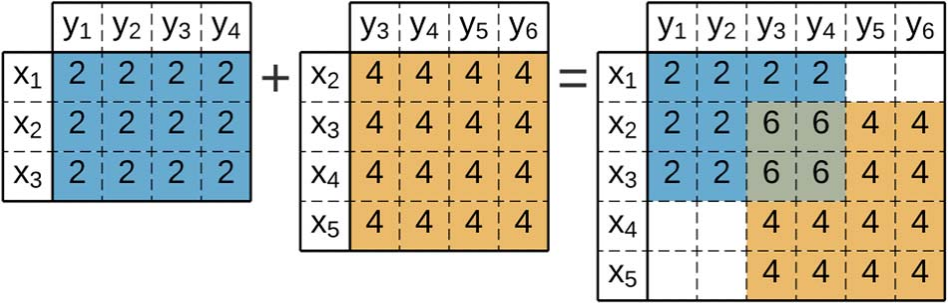
Plaid model with an **additive** cumulative function between two constant biclusters; plaid models are used in bioinformatics to represent the simultaneous participation of interacting biological processes.


When biclusters overlap, their interaction can be explained by plaid models, which decompose contributions for each overlapped bicluster [99]. Figure 15 illustrates the *additive plaid model* where the elements $a_{ij}$ of the data matrix are viewed as a sum of terms,

(1) \begin{eqnarray*} a_{ij} = \sum_{t=0}^{q}{\theta_{ijt}\rho_{it}\kappa_{jt}}, \end{eqnarray*}

where $\theta _{ijt}$ defines a contribution for each bicluster $B_{t}$, and both $\rho _{it}$ and $\kappa _{jt}$ are boolean values that state if the observation $x_{i}$ and attribute $y_{j}$ belong to the bicluster.

Biologically, plaid models reflect the participation of entities in several processes. Thus, simultaneously determining these contributions allows for improved interpretability of the underlying biological events [5]. In gene expression data, a plaid model considers the cumulative effect of processes and their interactions based on the expected score of each active process [99]. In network data, plaid models reflect the contributions of overlapping subgraphs, providing topologic insights into genes and protein–protein interactions in terms of core interactions and between- and within-pathway interactions [9].

Since the original definition of plaid models, discovering plaid structures has been expanded with additional definitions and new algorithms have been developed. We refer to Henriques and Madeira [5] for a comprehensive view of plaid biclustering models and an introduction to principles for discovering nontrivial interactions.

## Biclustering algorithm

The objective of a biclustering algorithm is to discover a biclustering solution. We discuss four characteristics of biclustering algorithms:

- **Algorithmic approach**;
- **Characteristics of the biclustering solution**;
- Concerns with **scalability**;
- Availability of **computational implementations**.

Table 4 summarizes the general characteristics of popular algorithms in the literature.

**Table 4:** Popular biclustering algorithms organized by their search heuristic; the labels in column 6, referring to the biclustering solution positioning, correspond to the labels in Figure 14

Search Heuristic		Algorithm	Bicluster	Biclustering Solution	Implementation
Category	Sub-Category		Coherence	Positioning	
Clustering-Based	Iterative Row and Column Clustering Combination	Coupled Two-Way clustering [100]	Constant [1]	I) [1]	N/A
		Interrelated Two-way Clustering [101]	Coherent [1]	D) and E) [1]	N/A
		Double Conjugated Clustering [102]	Constant [1]	D) and E) [1]	N/A
	One-Dimension Clustering	Possibilistic Spectral Biclustering Algorithm [103]	Coherent [18]	I) [18]	N/A
		Biclustering with SVD and Hierarchical Clustering [104]	Coherent [18]	I) [18]	N/A
Divide and Conquer		Direct Clustering [12]	Constant [1]	F) [1]	N/A
		Bimax [64]	Constant [64]	I) [64]	biclust
Greedy	Deterministic	Cheng and Church [2]	Coherent [1]	I) [1]	biclust
		HARP [105]	Constant [18]	I) [18]	N/A
		QUBIC [57]	Coherent [18]	I) [18]	r package
	Stochastic	ISA [55]	Coherent [18]	I) [18]	r package
		XMotifs [58]	Order Preserving [18]	I) [18]	biclust
	Nature-Inspired	EvoBexpa [106]	Coherent [18]	I) [18]	N/A
		Mitra and Banka [107]	Coherent [18]	I) [18]	N/A
		EBIC [108]	Several [108]	N/A	Github Repository
Exhaustive	Pattern Mining-based	CCC-Biclustering [11]	Order Preserving [11]	I) [11]	BiGGeSTS
		BicPAM [89]	Flexible [89]	I) [89]	BicPAMS
		DeBi [90]	Constant [90]	I) [89]	BicPAMS
		RAP [109]	Constant [109]	I) [89]	RAP website
		RIn-Close [110]	Coherent [110]	I) [110]	Github Repository
	Other	SAMBA [56]	Order Preserving [1]	I) [1]	EXPANDER
		BiBit [65]	Constant [65]	I) [65]	r package
Distribution Parameter Identification		FABIA [59]	Constant [3]	N/A	r package
		Spectral Biclustering [111]	Constant [3]	C) [1]	Scikit-learn
		Spectral Coclustering [112]	N/A	B) [1]	Scikit-learn
Essemble	Bagging	Hanczar and Nadif [113, 114]	N/A	N/A	N/A
		Aggarwal et Gupta [115]	N/A	N/A	N/A
	Boosting	Yin et Liu [116]	N/A	N/A	N/A
		Hanczar and Nadif [117]	N/A	N/A	N/A

### Algorithmic approach

Biclustering algorithms are categorized based on their search strategy. The **discovery** of biclusters describes how the algorithms discover the biclusters:

- **One at a time**: discover a single bicluster, or a single bicluster per iteration [2, 95, 99];
- **One set at a time**: discover a group of bicluster per iteration [12, 100, 118];
- **Simultaneous identification**: discover all biclusters at the same time [56, 58, 111].

The search **heuristic** describes how the biclustering algorithms search for the biclusters [18, 21, 22]. We consider the following categories: *Clustering-based*, *Divide and Conquer*, *Greedy*, *Exhaustive*, *Distribution Parameter Identification*, and *Ensemble*, expanded in the following sections.

#### Clustering-based

**Figure 16:**
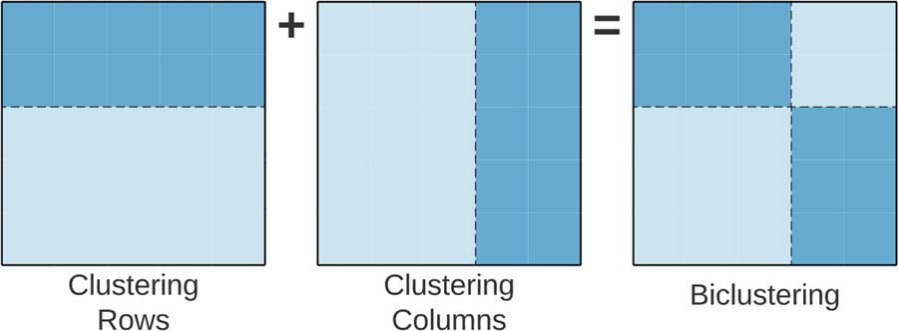
Clustering-based approaches combine results from traditional clustering algorithms with a strategy to group the second dimension; the results from the grouping in both dimensions result in a biclustering; in this illustrative approach, clustering is applied to both rows and columns of a data matrix (identifying two row clusters and two column clusters); combining the grouping results on a checkerboard biclustering structure with four biclusters.

Clustering-based algorithms use a traditional clustering approach with some heuristics to handle the second dimension. Figure 16 illustrates a naive approach for clustering-based biclustering.

There are two sub-categories for clustering-based biclustering: *Iterative Row and Column Clustering Combination*, and *One-Dimension Clustering Algorithms*.

**Iterative Row and Column Clustering Combination Algorithms** apply clustering algorithms twice: on the original matrix and the transposed matrix. The row and column clusters are then combined to obtain biclusters. Examples are the Coupled Two-Way clustering [100], Interrelated Two-way Clustering [101], and Double Conjugated Clustering [102].

**One-Dimension Clustering Algorithms** use a clustering algorithm on the matrix and then combine the clustering algorithm results using a heuristic to transform the original clusters into biclusters [103, 104].

#### Divide and conquer

**Figure 17:**
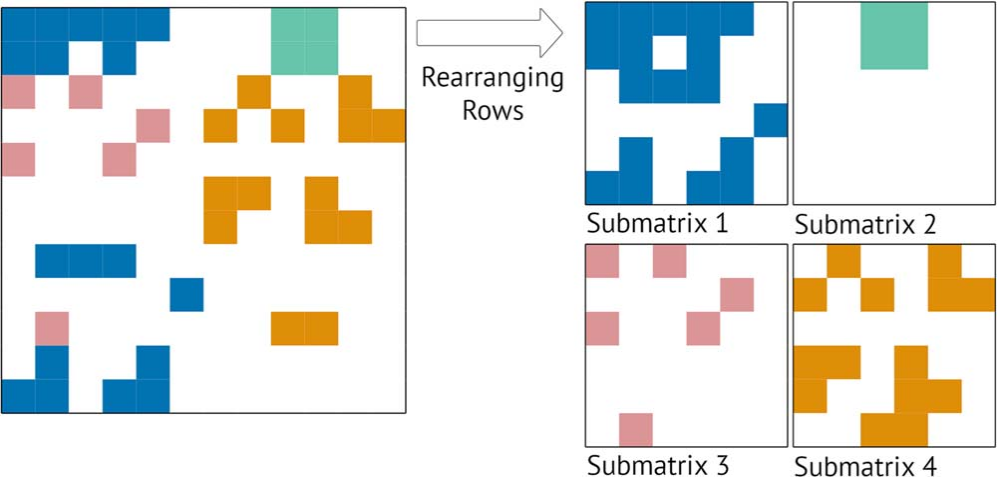
Divide and Conquer algorithms split the original data matrix into smaller instances; this example illustrates a splitting step, where the matrix is reordered into four sub-matrices (each represented by a color), and the same color means coherent values; admitting no additional splits in the matrix and no rows or columns are removed from the solution; the four sub-matrices correspond to four biclusters with higher homogeneity than the original matrix; further splits would be necessary to increase homogeneity by eliminating rows/columns with incoherent values in white elements.

Divide and Conquer algorithms, illustrated in Figure 17, divide the original data matrix into smaller submatrices. Examples of divide and conquer algorithms are Direct Clustering [12] and Bimax [64]. They aim to obtain biclusters with a higher homogeneity than the original dataset. The greater advantage of this approach is the execution speed. However, they are likely to miss good biclusters due to their rigid splitting approach.

#### Greedy

**Figure 18:**
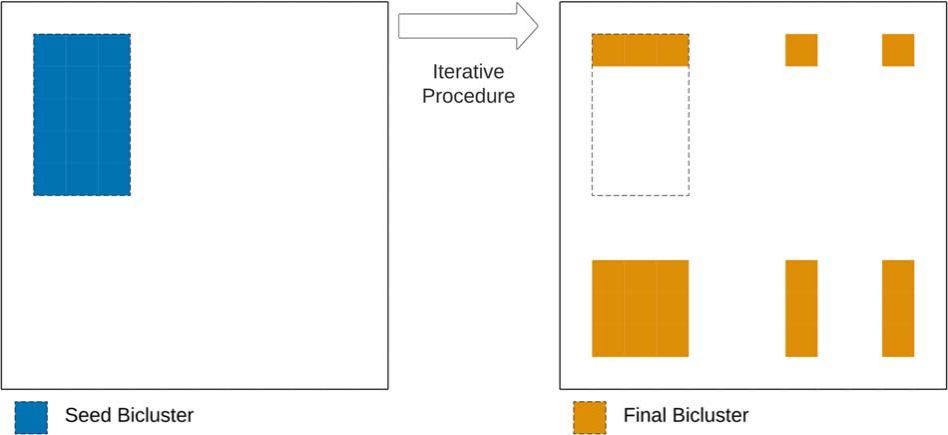
Greedy algorithms begin with an initial sub-matrix (seed bicluster) and use an iterative procedure to add and remove rows and columns from the biclusters to reach a local optimum given some merit function.

Greedy algorithms, illustrated in Figure 18, consider an initial solution and use an iterative procedure with a merit function to add and remove rows from the initial solution and discover a local optimum. This category is popular since it guarantees that the algorithms run in a reasonable and controllable time. While these algorithms risk being stuck on local optimum, they can be very fast.

We further divide this category into three sub-categories based on the specific search strategy: deterministic greedy, stochastic greedy, and nature-inspired [18].

**Deterministic Greedy** is a family of algorithms that use deterministic processes when adding and removing rows and columns from the initial solution [2, 105, 119].

**Stochastic Greedy** algorithms add a stochastic strategy to the iterative search of an optimal solution, guaranteeing nondeterministic results [55, 58]. This stochastic strategy is less prone to be stuck on local optimum since the user can always run the algorithm multiple times to guarantee optimal solutions, a strategy used in comparative studies [3].

**Nature-Inspired** are a category of search heuristic based on nature behaviors such as evolutionary computation, artificial immune systems, ants colony optimization, or swarm optimization [120]. Evolutionary computing is the most popular nature-inspired heuristic, with several proposed biclustering algorithms [106, 120–122]. We refer to Pontes *et al*. [18] for a review on nature-inspired biclustering.

#### Exhaustive

Exhaustive algorithms search for all possible biclusters in the matrix so that all the best biclusters are guaranteed to be discovered. Due to the high computational complexity of biclustering, these algorithms have performance drawbacks. There are some strategies to guarantee that the exhaustive algorithms run in a reasonable time. One is to limit the size of the biclusters, which guarantees that all biclusters will be discovered (given the size limit). Another is to search for all biclusters given a limited data domain such as binary data [65] or time series analysis [11].

A particular sub-category of exhaustive algorithms is **pattern mining-based** algorithms. Pattern mining techniques have emerged in biclustering analysis due to theoretical links between the two areas. Incorporating pattern mining techniques into the development of biclustering algorithms makes it possible to perform a flexible, exhaustive, and efficient exploration of the solution space [20, 110].

#### Distribution parameter identification

Distribution Parameter Identification algorithms assume a statistical model in the data matrix and then apply an iterative procedure on its parameters to minimize some criterion. This category includes a wide range of mathematical models such as *binary least squares* [123], *singular value decomposition* [111], and *statistic models* [59, 124].

#### Ensemble

Ensemble biclustering combines several biclustering algorithms’ to improve general performance. Ensemble biclustering approaches fall in either **bagging**, using the same algorithm on different partitions of the original matrix, then combining the results [55, 113–115, 125], or **boosting**, using different algorithms (or different configurations of the same) on the same data matrix [116, 117, 126]. Beyond algorithmic development, Hanczar and Nadif [127] reviewed the consensus functions on ensemble clustering and showed how to extend them to the biclustering context.

### Characteristics of the biclustering solution

A biclustering algorithm creates a biclustering solution. In this section, we consider using the characteristics of the biclustering solution to classify the algorithms.

A widespread criterion to analyze the algorithms is the **coherence** of the obtained biclusters. This is not a trivial task for two reasons: First, the presence of noise often implies that it is hard to estimate the specific bicluster coherence. Second, a biclustering solution can have biclusters with non-defined coherences. There are three strategies to analyze the coherency of a biclustering solution:

- Analyze the *optimization metric* of the algorithm (if the algorithm uses an optimization metric to guide the search). The algorithm is then classified based on the coherency that the metric is optimized to detect [18, 30];
- Consider the *parametrizable* coherency of the algorithm. If the users can parametrize the coherency assumption of the algorithm, then it is used to classify the solution [8, 89];
- Consider the results of *comparative studies* that analyzed the performance of biclustering algorithms on synthetic and real data to conclude how good algorithms are in data with different coherency assumptions [3, 20, 24–26, 64].

The **positioning** of the biclusters in the data matrix is also used to classify the algorithms. This classification is easier to use since the positioning of the biclusters is set during the algorithm development stage. Algorithms such as ISA [55], Bimax [64], and CCC-Biclustering [11] generate arbitrarily positioned biclusters, while Spectral Co-Clustering [112], Spectral Biclustering [111], and OPSM [95] have restrictions in row and column overlapping.

A relevant characteristic of biclustering algorithms is the user’s control of the *number* of generated biclusters. There are three types of algorithms:

- Some algorithms give *no control* to users regarding the number of biclusters to detect. An example is CCC-Biclustering, which has no parameters, and it is not possible to know how many biclusters will be discovered [11];
- A few algorithms admit an *indirect control* in the number of generated biclusters. An example is BicPAM, which uses iterations. If the number of iterations is increased, so will the number of mined biclusters [8, 89];
- Several algorithms have the number of generated biclusters as a *parameter*. However, this does not necessarily imply that the algorithm will always generate the number of requested biclusters since some algorithms will consider this parameter as an upper limit [2, 59].

### Scalability concerns

Biclustering is NP-complete due to the heavy combinatorial process of grouping subsets of observations with subsets of attributes [11, 97]. Its complexity, together with the growth in data volume in domains such as genomics, transcriptomic, and proteomic data, raises algorithmic challenges for the efficient and effective discovery of biclusters [128, 129].

In this context, the algorithms must guarantee a trade-off between the quality of the results and the quantity of used computational resources (avoiding the brute force process of discovering biclusters) [18, 21, 22]. There are three main strategies to guarantee computational efficiency: impose *data restrictions*, use *parametrization* to control heuristic search, and adopt *big data* strategies.

Some algorithms simplify the biclustering task using **data restrictions**. Examples are Bimax [64] and BitBit [65], which consider the specific case of binary data. Other examples are algorithms focused on time series analysis. Forcing the search for biclusters with contiguous columns simplifies the combinatorial process, allowing polynomial [73, 74] or even linear [11] execution times.

A popular strategy is to control the execution time in the **parametrization** of the algorithm, either by controlling the *number of iterations* parameter (present in greedy algorithms such as ISA [55], XMotifs [58], and Cheng and Church [2]), *error control* parameters (used by algorithms such as FABIA [59], BicPAM [89], and QUBIC [57]) or by *limiting the bicluster size* (used by BicPAM [89], Bimax [64], and Spectral Biclustering [111]).

Finally, there are algorithms developed considering **Big Data Analysis** strategies, either by adapting traditional algorithms [130, 131], or by developing new algorithms specifically for this scenario [132, 133]. Three strategies are used: Parallel Computing, GPUs, and the Map Reduce Programming paradigm [128, 129]. Table 5 lists biclustering approaches for Big Data analysis.

**Table 5:** Biclustering implementations specifically developed for big data analysis

Programming Strategy	Description	Biclustering algorithms	Original Algorithm
Parallel Computing	Divides the execution of the workflow into multicore processors or clusters of computers	PBiclustering [134]	New approach
		Cheng_Church [130]	Adapted from [2]
		runibic [135]	Adapted from [136]
		ParBiBit [131]	Adapted from [65]
		EBIC.JL [137]	Adapted from [132]
		ScalaParBiBit [138]	Adapted from [131]
Using GPUs	The algorithms use the GPU of a computer instead of the CPU.	NMF [139]	Adapted from [140]
		FLOC [141]	Adapted from [142]
		GBC [143]	Adapted from [144]
		MMPC [145]	New approach
		CCS [146]	New approach
		EBIC [132]	New approach
		CUBiBit [147]	Adapted from [131]
		gBiBit [148]	Adapted from [131]
Map Reduce	Makes use of the Map Reduce programming paradigm to process data in parallel and distributed systems.	DisCo [149]	New approach
		NMF [150]	Adapted from [140]
		BiTM-MR [133]	New approach

### Computational implementations

Most of the popular implementations for biclustering, listed in Table 6, are developed in *Python*, *R*, or *Java* programming languages, and some software with *GUIs* are also available.

**Table 6:** Computational implementations for biclustering

Name	Description	Available Algorithms	Aditional Information	References
Scikit-learn	Part of the Python machine learning library scikit-learn.	Spectral Co-clustering [112], Spectral Biclustering [111]	One external measure is implemented.	[151]
Biclustlib	General purpose Python library.	BCCA [152], BiBit [65], Cheng and Church [2], LAS [153], Plaid [123], xMotifs [58], FABIA [59], Spectral Biclustering [111], Bimax [64], ISA [55], BCC [124], OPSM [95], QUBIC [57], RInClose [110]	Includes data collection and external evaluation measures.	[3]
biclust and BiclustGUI	General purpose R package and associated GUI.	Bimax [64], Cheng and Church [2], Plaid [123], Questmotif [58], Xmotifs [58], Spectral Biclustering [111]	Includes methods for data preprocessing, visualization, and validation of bicluster solutions.	[154]
BicPAMS	GUI and Java API focused on pattern-based biclustering.	BicPAM [89], BicNET [9], BicSPAM [60], BiC2PAM [6], BiP [5], DeBi [90], BiModule [155]	Includes data collections, integrated methods for data preprocessing, and methods for visualization of biclusters.	[8]
BiGGeSTS	GUI focused on time series biclustering.	CCC-Biclustering [11], e-CCC-Biclustering [73], LateBiclustering [74], CC-TSB [156]	Includes methods for data preprocessing, biclustering analysis, and post-processing methods. Includes variants of CCC-Biclustering to detect more complex patterns.	[36]
BicAT	GUI to analyze gene expression data with both biclustering and clustering algorithms.	Bimax [64], CC [2], ISA [55], xMotifs [58], OPSM [95]	Includes preprocessing and post-processing facilities.	[23]
EXPANDER	GUI to analyze gene expression and next-generation sequencing data.	SAMBA [56]	Includes clustering algorithms, tools for gene expression analysis, and visualization.	[157]
Biorithm	Tools to analyze data in molecular systems biology.	xMotifs [58]	Includes BiVoC, a package for bicluster visualization.	
Bicoverlapper	Tools focused on visualization of biclusters, transcription regulatory networks, and gene annotations.	Bimax [64], Cheng and Church [2], Plaid [123], Xmotifs [58]		[158, 159]
G-Bic	Synthetic dataset generator for biclustering, focused on producing synthetic data with biclustering solutions.			[34]

## Evaluation measures

In this section, we present techniques to analyze and interpret biclustering solutions. There are five approaches to evaluate the results of biclustering algorithms: *Visualization techniques*, *External metrics*, *Internal metrics*, *Statistical significance metrics*, and *Problem-specific evaluation*.

### Vizualization techniques

**Figure 19:**
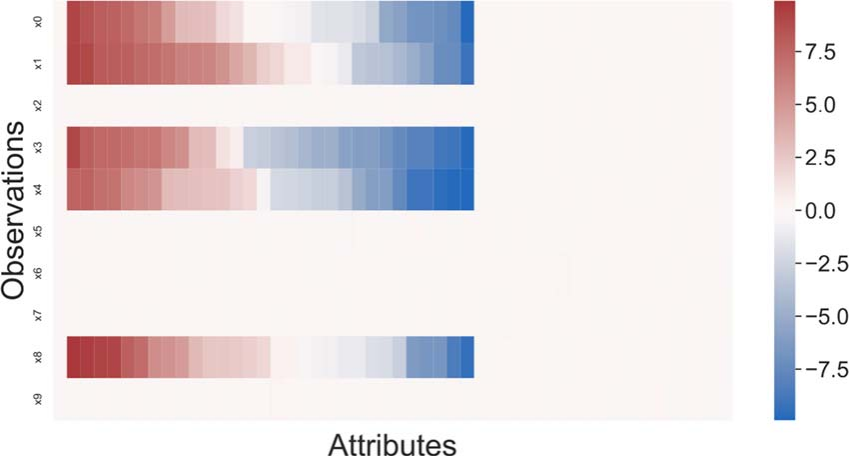
Heatmaps are a technique to visualize biclusters, which is particularly effective if the biclusters have values clearly distinguished from the background and can be represented contiguously; this figure represents a heatmap of a single synthetic bicluster in a dataset without background.

**Figure 20:**
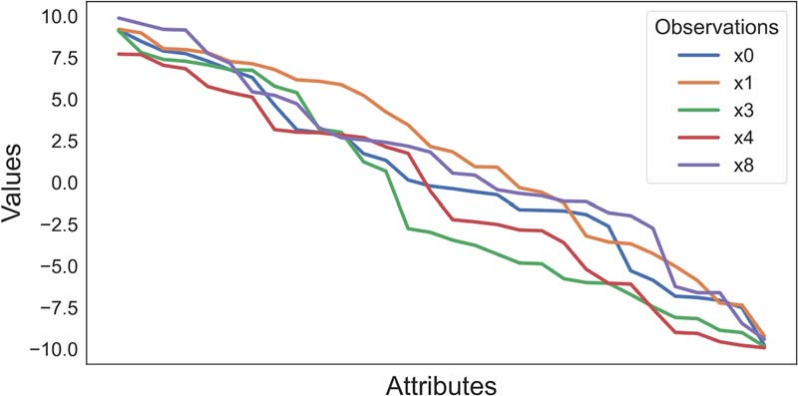
Parallel coordinates are a representation of a bicluster that allows an easy visualization of the bicluster’s pattern; this figure illustrates the bicluster previously represented in Figure 19.

Compared with visually interpreting clustering results, biclustering faces additional challenges due to the simultaneous grouping of rows and columns, the overlapping, and the tendency of algorithms to generate a high number of biclusters [158]. There are techniques to visualize a single bicluster and the entire biclustering solution. Considering the visualization of a single bicluster, **heatmaps** (Figure 19) and **parallel coordinates** (Figure 20) are popular options [158, 160]. While parallel coordinates are only legible to visualize biclusters with a small number of observations, they are good options for identifying patterns such as symmetries and time lags [8, 69, 74]. Heatmaps illustrate larger biclusters more easily. However, their use depends on the choice of color. In both methods, a shuffling of rows/columns could be needed for an improved visualization [69, 161, 162].

**Figure 21:**
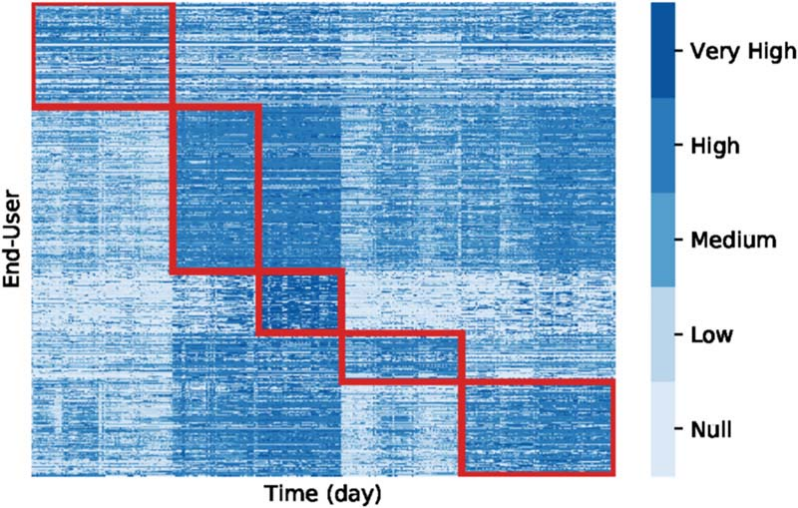
If the biclustering solution has little to no overlap between biclusters, heatmaps are an efficient approach to represent a biclustering solution visually; in this figure, adapted from Silva *et al*. [69], heatmaps represent a biclustering solution consisting of five biclusters.

Considering a biclustering solution, the most popular options for biclustering visualization are heatmaps [69, 163–165]. Visualizing a biclustering solution would resemble structures from Figure 14 in ideal conditions. An example is Figure 21, which shows a biclustering solution resembling exclusive row and column biclusters. While the visualization of nonoverlapping biclusters in a heatmap is more straightforward, the visual representation of overlapping biclusters has geometrical limitations [158]. BiVoc [166] implements a method to better show biclusters in a two-dimensional layout to reveal overlaps and relationships. However, since this approach uses rows/column duplication, it can lead to potential ambiguities and misinterpretations [158].

**Figure 22:**
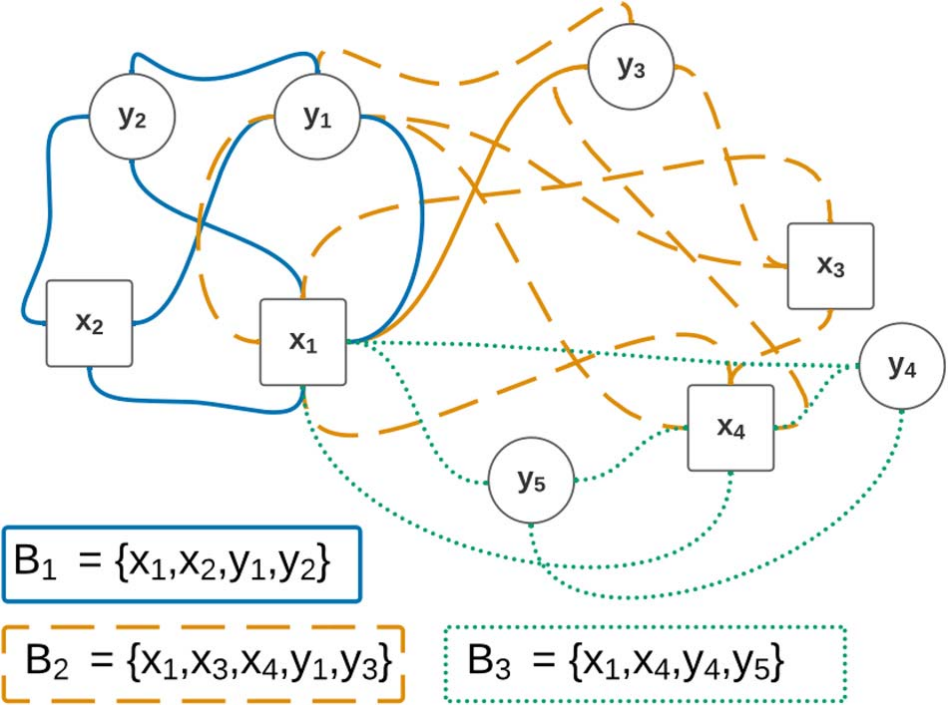
Graphs can be used to represent biclustering solutions in a graph consisting of labeled edges; this figure represents a biclustering solution made of three biclusters in a graph; the presence of more than one edge between two nodes implies overlapping; for instance, $B_{1}$ and $B_{2}$ overlap in $(x_{1},y_{1})$.

**Figure 23:**
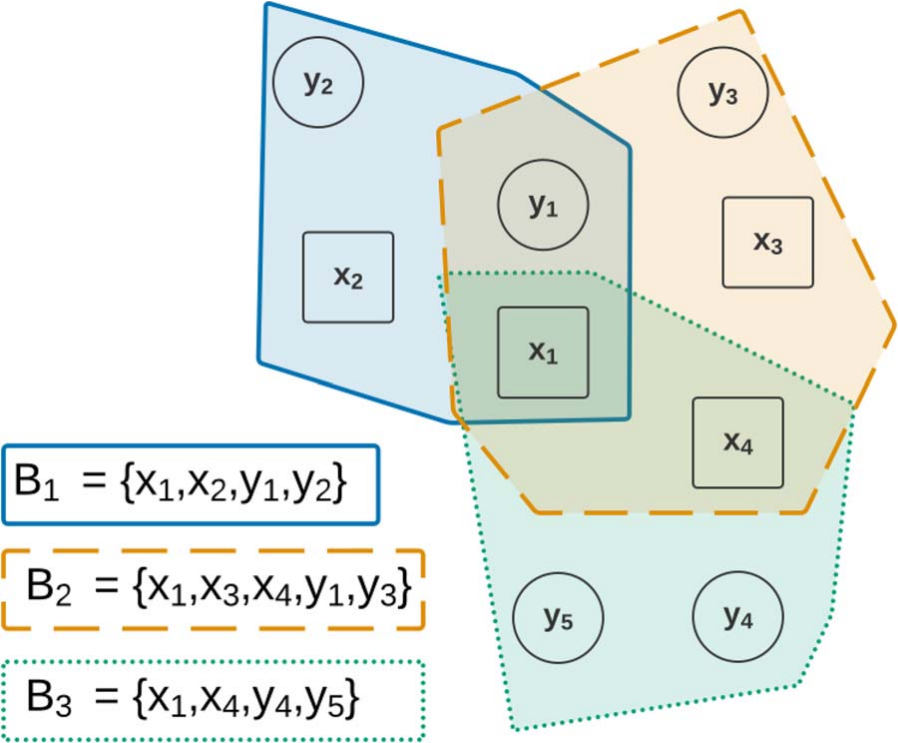
As an alternative to edges in a graph, the shaded areas can be used to represent the biclustering solution; this figure represents the same biclustering solution as Figure 22, and the overlapping of areas implies the overlapping of biclusters.

Biclustering solutions can be represented using graphs with observations and attributes represented as nodes [158, 167, 168]. Biclusters are then represented either by labeled edges in a graph (Figure 22) or by a shaded area (Figure 23).

**Figure 24:**
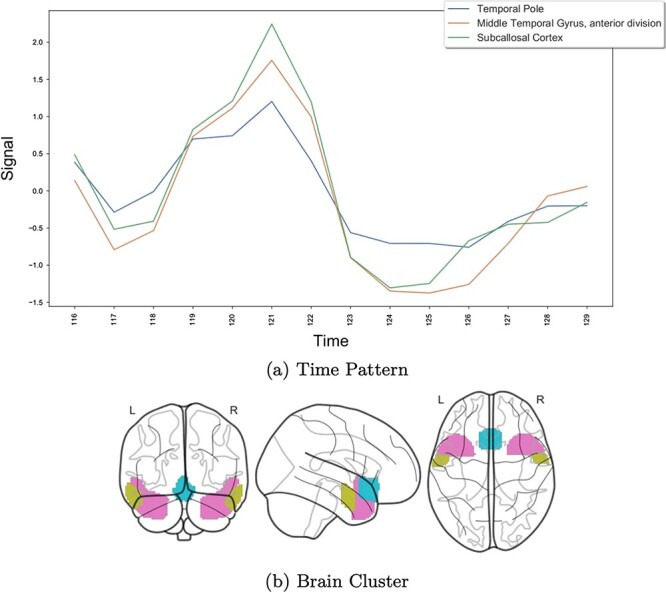
If the biclusters have spatial or temporal information, this can be used to represent biclusters; this figure, adapted from Castanho *et al*. [26], shows a spatio-temporal bicluster; the temporal part is used to show the temporal pattern of the signal in (a), while (b) represents the spatial part showing the brain regions with similar activity identified by the bicluster.

A particular case for biclustering visualization is spatio-temporal data analysis. In these cases, it is usual to represent the biclusters on a map [68], as illustrated in Figure 24, showing the brain regions corresponding to a bicluster.

### External metrics

External metrics, extrinsic or similarity evaluation metrics, are used when ground truth is known. They aim to quantify how well a biclustering algorithm retrieves the true biclustering solution. The presence of multiple biclusters in the matrix and overlapping add complexity to the measure of how well can a biclustering algorithm retrieve a solution [28].

These metrics require two biclusterings, a discovered biclustering $\mathcal{B}$ and a reference $\dot{\mathcal{B}}$ biclustering (considered as ground truth). An external metric $S(\mathcal{B},\dot{\mathcal{B}})$ compares the similarity of the two biclusterings. These metrics are often obtained with variations of the F-Measure [169] or Jaccard-based scores [170]. Horta and Campello [28] discussed eight relevant properties for these metrics and how current measures verify these properties, summarized in Table 7.

**Table 7:** Properties that external measures used to evaluate biclustering solutions, $S(\mathcal{B},\dot{\mathcal{B}})$ should have according to Horta and Campello [28]

Property	Definition	Metrics that verify the property
Size of Spurious Biclusters	The measure should be sensitive to an equally sized but noisier biclustering solution.	$S_{rnia}$ [171], $S_{ce}$ [171], $S_{wjack}$ [172], $S_{wdic}$ [172], $S_{e}$ [24], $S_{csi}$ [28], $S_{ebc}$ [28]
Coverage	The measure should be sensible to incomplete solutions.	$S_{rnia}$ [171], $S_{ce}$ [171], $S_{fabi}$ [59], $S_{csi}$ [28], $S_{ebc}$ [28]
Non-intersecting Area	The measure should be sensible to a noisier biclustering solution.	$S_{rnia}$ [171], $S_{ce}$ [171], $S_{e}$ [24], $S_{csi}$ [28], $S_{ebc}$ [28]
Multiple Coverage	The measure should penalize solutions made of several real biclusters.	$S_{prel}$ [64], $S_{prec}$ [64], $S_{ce}$ [171], $S_{l\&w}$ [173], $S_{stm}$ [29], $S_{wjac}$ [172], $S_{wdic}$ [172], $S_{fabi}$ [59], $S_{ay}$ [174], $S_{erel}$ [25], $S_{erec}$ [25], $S_{csi}$ [28], $S_{ebc}$ [28]
Repetitive Biclusters	The measure should penalize a solution with repetitive biclusters.	$S_{rni}$ [171], $S_{ce}$ [171], $S_{csi}$ [28], $S_{ebc}$ [28]
Symmetry	The measure should verify $S(B_{a},B_{b})=S(B_{b},B_{a})$ for any $B_{a}$, $B_{b}$ solutions.	$S_{rnia}$ [171], $S_{ce}$ [171], $S_{fabi}$ [59], $S_{csi}$ [28], $S_{ebc}$ [28]
Homogeneity	The measure should penalize fewer homogeneity solutions.	$S_{csi}$ [28], $S_{ebc}$ [28]
Conditions for Maximum	The measure must have the maximum values if the discovered biclustering fully equals the real one.	$S_{ce}$ [171], $S_{fabi}$ [59]

External evaluation metrics have been intensively used in comparative studies to evaluate the capacity of a biclustering algorithm under controlled circumstances, recurring to synthetic data [3, 20, 24, 25, 64].

### Internal metrics

Internal metrics, also known as quality or coherence metrics, estimate the intrinsic homogeneity of a biclustering. Depending on the study, these metrics are used to either guide the stochastic search for the biclusters or to compare the homogeneity of biclustering on real data. Pontes *et al*. [30] conducted a survey analysis of internal evaluation metrics and a comparative study of their capacity to detect different coherency patterns. Their results are summarized in Table 8.

**Table 8:** Internal metrics are used to evaluate the internal quality of biclusters; Pontes *et al*. [30] evaluated the capacities of several measures on different coherence assumptions

Metric	Pattern
Variance [12]	Constant
Row and Column Variance [119]	Constant
Mean Squared Residue [2]	Additive Coherent
Scaling Mean Squared Residue [119]	Multiplicative Coherent
Pearson’s Correlation Coefficient [175]	Coherent
Average Correlation [176]	Other
Sub-Matrix Correlation Score [104]	Other
Average Correlation Value [177]	Coherent
Average Spearman’s Rho [178]	Other
Spearman’s Biclustering Measure [179]	Coherent
Maximal Standard Area [180]	Coherent
Virtual Error [181]	Coherent
Virtual Error Transposed [182]	Coherent

### Statistical significance

Statistical significance evaluates how relevant the bicluster is in the data matrix considering a background of random, noisy data. Since good levels of homogeneity appear by chance in a data matrix, statistical metrics allow a reduction of the occurrence of false positives.

Similar to internal metrics, sophisticated statistical significance metrics require the assumption of the coherence of biclusters. For a study on measures to evaluate the statistical significance of biclustering solution, we refer to Henriques and Madeira [31] that integrated scattered studies on the statistical significance of biclustering solutions with new proposals to evaluate the statistical significance of biclusters with different coherency types.

### Problem-specific evaluation

An alternative to the previous evaluation metrics is to consider the specificities of each application domain, relying on domain expertise [64]. In biclustering, a popular metric to analyze results from gene expression data is their biological significance [28].

Suppose a set *Genes $\times $ Conditions* matrix. In this case, it is possible to evaluate the genes in a bicluster using databases such as the Gene Ontology (GO) [183, 184], or the Kyoto Encyclopedia of Genes and Genomes (KEGG) [185] to obtain a *P-value* indicative of the randomness of the found bicluster [36].

Due to the historical relationship between biclustering and the context of gene expression data analysis, biological measures have been intensively used either by comparison studies [3, 20, 24, 25, 64] or new algorithm proposals [5, 11, 59, 73, 74, 89] to compare algorithm performance. This approach has two significant disadvantages. First, it is specific to the gene expression data context and therefore not valid for any other context. Additionally, it entirely disregards the *conditions* dimension and analyzes results exclusively considering the genes in a bicluster.

## Applications

Following the development of Cheng and Church algorithm, applied to gene expression data, biclustering has demonstrated significant potential in bioinformatics due to its flexibility to discover biological modules. This is particularly useful in tasks such as the analysis of biological networks [186] and assessing molecular units involved in cellular functions [5, 90].

A previous study by Xie *et al*. [7] analyzed the biclustering literature, concluding that around $40\%$ of the studies published between 2012 and 2017 were applicational. A recent *PubMed* search indicates that, in the last 5 years, the proportion of applicational studies is now around $60\%$ of the literature (Application: $57\%$, Algorithmic: $32\%$, Software: $5\%$, Comparative: $2\%$, Survey: $1\%$, Measure: $1\%$). These results highlight the growing focus on applicational studies of biclustering compared with other types of studies, such as algorithm development.

In this section, we approach the challenge of applying biclustering in data analysis. First, in **Data Mining Tasks**, we discuss the use and application of biclustering in conjunction with other data mining tasks. Next, in **Criteria to Select a Biclustering Algorithm**, we explore how to identify the most adequate biclustering algorithm for a given applicational scenario. In **Interpretability of a Biclustering Solution**, we examine the challenges involved in analyzing biclustering results to extract actionable insights. Finally, in **Application Domains**, we categorize and discuss various applications of biclustering.

### Data mining tasks

Biclustering is an unsupervised learning approach used to discover patterns hidden as sub-matrices within a data matrix. The evaluation and interpretation of these sub-matrices considers both quantitative and qualitative measures. Biclustering results are often related to other data mining tasks such as *Clustering*, *Pattern Mining*, *Classification*, *Triclustering and N-way Clustering*, and *Graph Mining*. In this section, we compare biclustering with these approaches. Table 9 summarizes the conclusions of this section by highlighting the similarities between biclustering and these other data mining tasks and the unique opportunities that biclustering presents in this context.

**Table 9:** Comparison between biclustering and other data mining tasks

Data mining approach	Similarities	Differences	Opportunities
Hard clustering	Both approaches discover groups of similar observations.	Biclustering algorithms discover (possibly overlapping) clusters with broader definitions of similarity (local versus global patterns).	Biclustering automatically selects the relevant attributes for each cluster; Algorithms that force exclusive rows discover nonoverlapping groups.
Soft clustering	Both approaches discover structures where an observation can belong to more than one group.	Biclustering algorithms do not assign probabilities of an observation belonging to a group.	Most biclustering approaches allow overlapping structures with broader definitions of similarity.
Pattern Mining	Both approaches discover overlapping structures with a potentially interesting pattern.	While pattern mining focuses on discovering patterns and association rules, biclustering discovers sub-matrices. The analysis of the pattern in the matrices is a post-processing step.	Biclustering generalizes pattern mining, discovering broad patterns in both homogeneous or heterogeneous matrices.
Classification	Both biclutering and associative classifiers learn from subspaces in data.	Biclustering is unsupervised, in contrast to classifiers.	Biclustering can be used to learn new features that a classifier can use.
Triclustering	Is a generalization of biclustering to 3-way data (observation-attributes-context).	Are applied to tensorial datasets, while biclustering is applied to data matrices.	Inherits definitions from biclustering, benefiting from the fact that biclustering research can be generalized to tensorial data.
N-way Clustering	Is an additional generalization of biclustering or triclustering for N-dimensional datasets.	Same as triclustering.	Same as triclustering.
Graph Mining	Biclustering can be seen as searching cliques in bipartite graphs.	Graph Mining algorithms are usually applied to graph structures, while biclustering requires a data matrix (that can represent a graph).	Biclustering algorithms can be used to discover dense regions in a network.

#### Clustering

**Figure 25:**
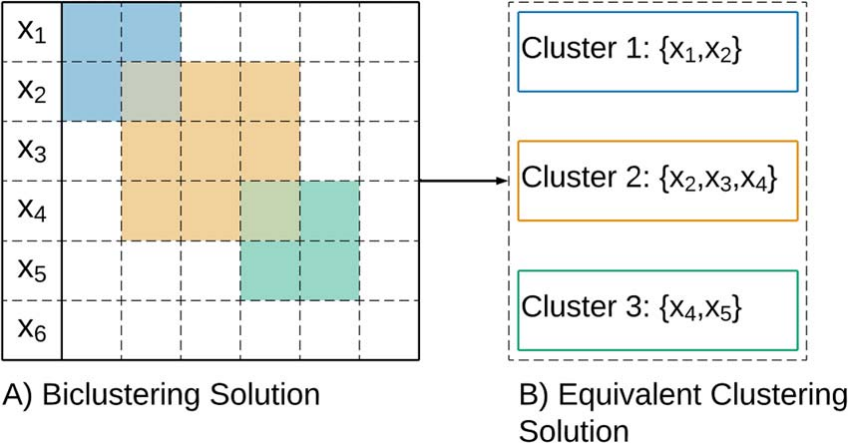
Biclustering performs grouping in both rows and columns of a data matrix; therefore, it can be seen as a form of (soft) clustering that automatically identifies the relevant attributes for the grouping between observations.

The clustering of observations, also known as row clustering, is a technique based on grouping similar observations [187]. As a generalization of clustering, biclustering can be used for clustering, disregarding the grouping on the second dimension and focusing the analysis on the grouping between observations, as illustrated in Figure 25. Similar conclusions can be extracted for attribute clustering, grouping similar attributes.

Compared with clustering, biclustering algorithms automatically select the relevant columns for each cluster, with no need to use dimensionality reduction techniques. Since a bicluster is typically smaller than a cluster (as it can have a smaller number of columns compared with clustering), each bicluster has an easier interpretation than a cluster. Additionally, a bicluster is typically more homogeneous, capturing specific coherences that clustering fails to detect [26]. Depending on the structure of each biclustering solution obtained by the algorithms, it is possible to use biclustering for both hard and soft clustering. While hard clustering corresponds to traditional clustering algorithms that divide observations or observations into disjoint sets, soft-clustering admits overlapping between observations [188, 189]. The closest biclustering algorithms to hard clustering are the ones that detect *exclusive rows* [101, 102, 112]. Generic arbitrarily overlapping biclustering algorithms can be used to obtain these soft clusters.

#### Pattern mining

Pattern mining involves the discovery of interesting patterns. While clustering groups similar observations, pattern mining algorithms focus on locating specific relationships between attributes [190, 191]. Pattern mining is conceptually close to biclustering, with the discovered patterns being essentially biclusters in a transaction data matrix [20].

The theoretical link between pattern mining and biclustering has enabled the incorporation of pattern-based principles in the biclustering literature. This has contributed to *(1)* improved interpretability of biclustering results, since a bicluster is described by its pattern, *(2)* improved principles of algorithmic development, *(3)* new strategies for determining the statistical significance of biclustering solutions, and *(4)* the application of classification principles [20, 31, 38].

#### Classification

**Figure 26:**
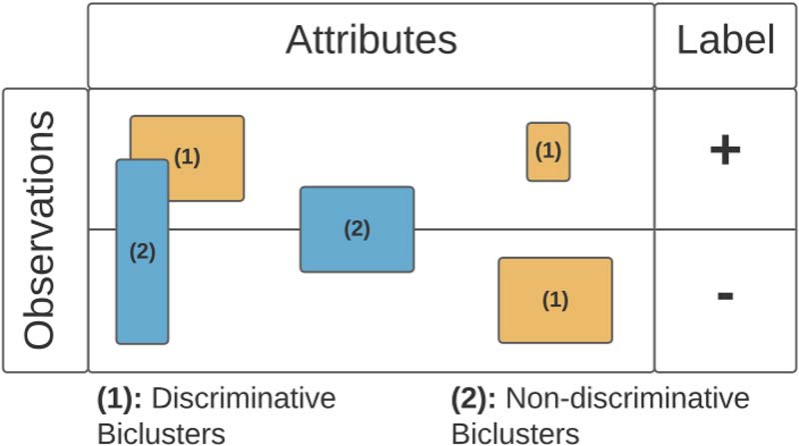
A bicluster represents a characteristic of part of a class (identified by a label in the data matrix), so it is possible to have discriminative power; if a discriminative bicluster identifies a class, it has the potential for classification.

While biclustering is an unsupervised data mining technique, its ability to discover local patterns with nonconstant coherences raises the potential for its use in supervised tasks. The principle assumes that a biclustering solution will have biclusters with *discriminative power*, where observations belong to only one class [38]. Figure 26 illustrates the concept of a discriminative bicluster.

**Figure 27:**
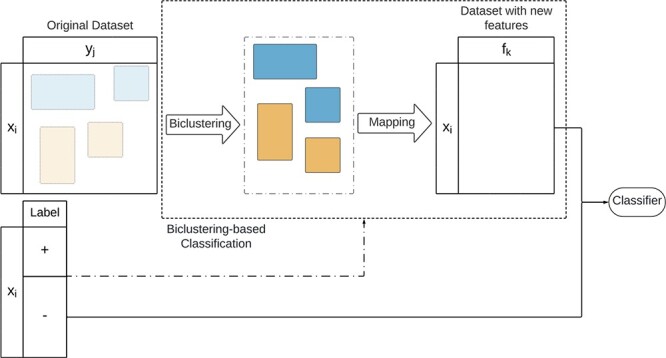
Biclustering-based classification is based on integrating information from a biclustering solution into a new set of features then used for classification; the search for the biclusters is unsupervised, but the labels can be used during preprocessing or post-processing to improve the discriminative power of the biclustering solution.

Biclustering-based classification aims to use information from a biclustering solution to enhance the performance of traditional supervised approaches. This approach, illustrated in Figure 27, applies a biclustering algorithm to the data matrix. Ground truth can be used to improve the search for discriminative biclusters, either by applying biclustering separately on each class-conditional data partition or by post-processing the dataset to filter non-discriminative biclusters [38, 192]. After discovering the solution, a mapping strategy computes new features from the biclustering solution. These features are then used to train a classic classifier. We refer to Henriques and Madeira [38] for a comprehensive view of biclustering-based classification.

There are three strategies to compute features from the extracted biclusters: *Biclusters as features*, *Patterns as features*, and *Rule-based features*. Biclustering-based classification strategies are summarized in Table 10.

**Table 10:** Approaches for biclustering-based classification

Approach	Study	Biclustering Algorithm	Classifier	Objective	Other
Biclusters as features	Carreiro *et al*. [70]	CCC-Biclustering [11]	k-Nearest Neighbors	Classify the response of multiple sclerosis patients to the standard treatment of Interferon-$\beta $.	
	Carreiro *et al*. [71]	CCC-Biclustering [11]	k-Nearest Neighbors	Classify the response of multiple sclerosis patients to the treatment of Interferon-$\beta $	Use hierarchical clustering to create meta-biclusters [113] (clusters of biclusters)
	Matos *et al*. [192]	BicPAM [89]	Random Forest	Characterize groups of amyotrophic lateral sclerosis patients based on disease progression.	
	Soares *et al*. [76]	TCtriCluster [78]	Random Forest	Predict the need for non-invasive ventilation in amyotrophic lateral sclerosis.	
Patterns as features	Soares *et al*. [78]	TCtriCluster [78]	Naive Bayes, SVM with Gaussian kernel, XGBoost, and Random Forests	Predict 5 clinical endpoints for amyotrophic lateral sclerosis patients	
	Soares *et al*. [77]	TCtriCluster [78]	Naive Bayes, SVM with Gaussian kernel, XGBoost, and Random Forests	Predict the need for non-invasive ventilation in amyotrophic lateral sclerosis	Applies TCtriCluster separately for longitudinal and static data.
	Patricio *et al*. [193]	BicPAMS [8]	Naive Bayes, KNN, SVM, Decision Tree, Random Forest, XGBoost	Predict treatment response in Hodgkin’s Lymphoma	
	Zhang *et al*. [194]	N/A	Fuzzy inference	Classify breast tumors.	
	Huang *et al*. [195, 196]	N/A	AbaBoost	Classify breast tumors.	
Rule-based features	Huang *et al*. [197]	CCA [2]	Adaboost	Identify gases in air quality monitoring systems.	
	Henriques and Madeira [38]	BicPAMS [8]	rule-based classifiers	Evaluates their classification approach on 4 gene expression datasets and 8 clinical databases.	

**Figure 28:**
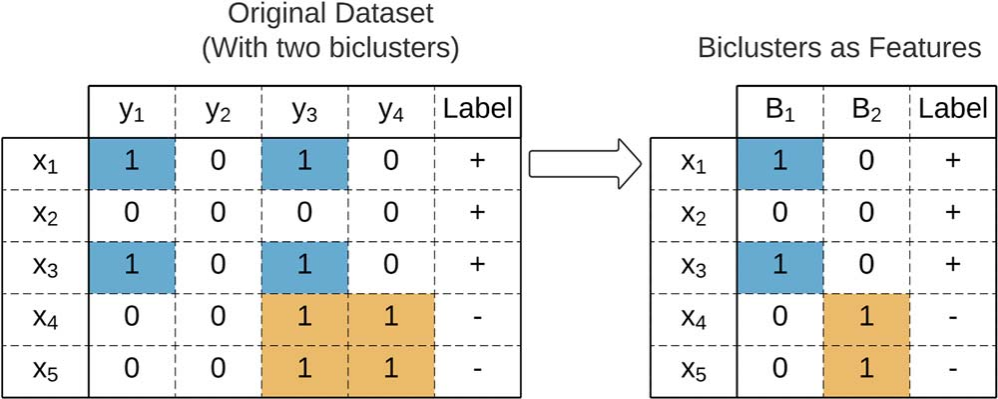
Biclusters as features: $B_{2}$ is a discriminative bicluster useful for classification.

**Biclusters as features** is illustrated in Figure 28. After discovering the biclusters, binary features are constructed, identifying the presence of a bicluster in each observation.

**Figure 29:**
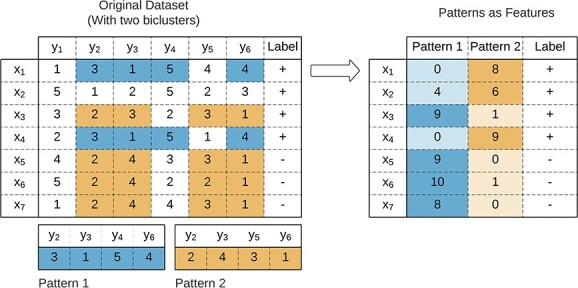
Patterns as features: uses the similarity between each observation and the computed patterns of the extracted biclusters for classification; for illustrative purposes, we assumed the row pattern of the bicluster as a signal (computed with the bicluster’s mode), and the Manhattan distance was used to estimate the distance between each observation and the bicluster.

**Patterns as features** begins by computing the biclustering solution and identifying each bicluster pattern. The similarity between each observation and the calculated pattern is used for classification. This strategy, illustrated in Figure 29, is not original from biclustering since several pattern mining studies use it for classification [77, 198–200].

Finally, **Rule-based features** are a strategy that combines the discovery of discriminative biclusters, with a pattern-based approach [8, 20, 197] with associative principles [201–204] and rule-based classifiers [202, 205, 206], using association rules to filter non-discriminative patterns and penalize similar patterns. Inspired by these rule-based features, *FleBIC* is the first associative classifier integrating biclustering during its pipeline [38].

#### Triclustering and N-Way clustering

The last years, there has been an increase in the use of tensorial datasets due to their ability to explain multivariate events such as biological responses, social interactions over time, urban dynamics, and complex geophysical phenomena [207]. These datasets are characterized by $N$ observations, $X$, $M$ attributes, $Y$, and $L$ contexts, $Z$.

**Figure 30:**
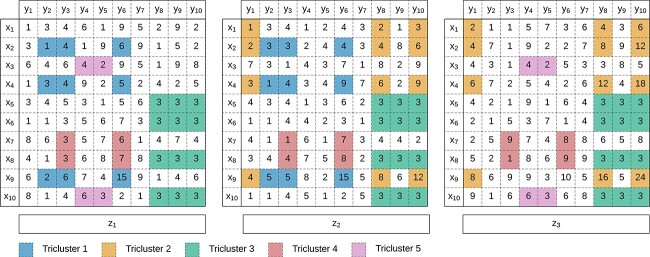
Tensorial dataset with 10 observations, 10 attributes, and 3 contexts with five nonoverlapping triclusters; figure adapted from Lobo *et al*. [96].

Triclustering, illustrated in Figure 30, is a generalization of biclustering whose task is discovering three-way subspaces and has applications in biological, biomedical, and social data [97,208].

Triclustering inherits theoretical definitions from biclustering, which is evident in metric and algorithm development. For evaluation metrics, several metrics used in biclustering were adapted for triclustering, including variance [97], mean square residue [209], virtual error [77], and statistical significance [210]. In terms of algorithm development, a popular strategy for discovering triclusters begins by slicing the dataset into two-way datasets, applying biclustering to each context, and then considering some heuristic to join the results [76, 87, 211, 212].

*N-Way Clustering* is a generalization from subspace clustering such that biclustering and triclustering are 2-clustering and 3-clustering. Contributions to N-Clustering are scarce due to optimization issues and problems formulating.

A comprehensive survey on triclustering and N-way clustering is presented by Henriques and Madeira [97]. Table 11 highlights application-based studies of triclustering. Triclustering-based classification is also possible [76–78].

**Table 11:** Application scenarios for triclustering

Domain	Sub-domain	Illustrative Biclusters
Biological Data	Gene Expression	*gene* $\times $ *sample* $\times $ *time* [213, 214]
		*disease* $\times $ *gene* $\times $ *GO term* [215]
		*gene* $\times $ *sample* $\times $ *regulator* [216]
		*gene* $\times $ *sample* $\times $ *organism* [217, 218]
	Biological Networks	*node* $\times $ *node* $\times $ *time data* [219]
		*source* $\times $ *node* $\times $ *node* [220]
Biomedical Data	Physiological	*individuals* $\times $ *features* $\times $ *time* [221]
		*patients* $\times $ *electrodes* $\times $ *time* [222]
		*patients* $\times $ *brain parcels* $\times $ *time* [87]
	Clinical	*patients* $\times $ *features* $\times $ *time* [76–78, 223]
		*patients* $\times $ *symptoms* $\times $ *time* [224]
Other Domains	Economy	*stock* $\times $ *ratio* $\times $ *item* [225, 226]
		*indicator* $\times $ *covariate* $\times $ *time* [207]
		*country* $\times $ *country* $\times $ *time* [207]
	Geophysical	*latitude* $\times $ *longitude* $\times $ *attribute* [227]
		*location* $\times $ *location* $\times $ *time* [228]
		*location* $\times $ *attribute* $\times $ *time* [229, 230]
	Crime	*regions* $\times $ *crimes* $\times $ *time* [231]
	Text Mining	*source* $\times $ *user* $\times $ *tag* [232, 233]

#### Graph mining

**Figure 31:**
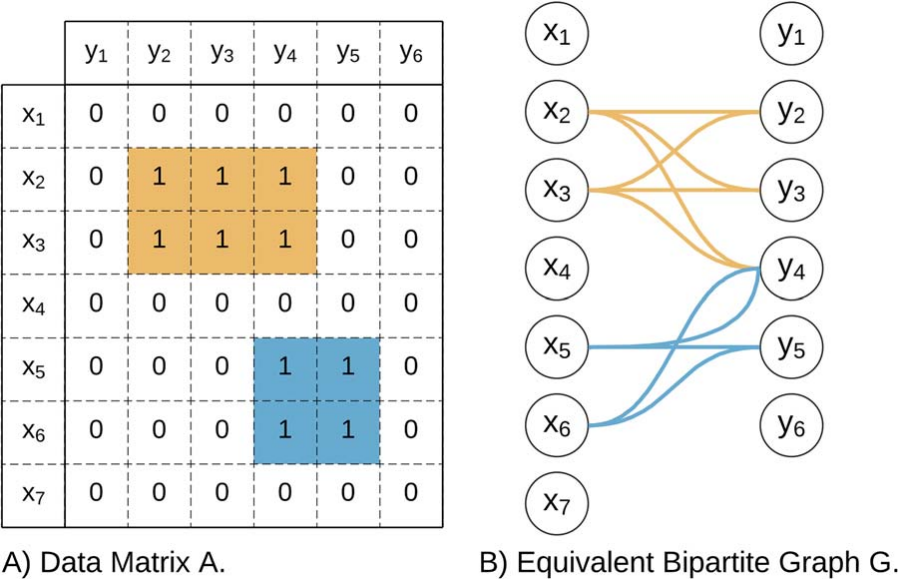
Graph view of biclustering; the **A**) biclusters (blue and orange) in a data matrix are redefined as **B**) cliques in a bipartite graph.

Biclustering can be applied to discover relationships between entities in different types of networks [9], even though it is not directly an algorithm for graph mining. Since the graph can be represented as an adjacency matrix, biclustering algorithms can discover coherent modules in binary and weighted networks [7, 234]. In biclustering, a data matrix can be seen as a weighted bipartite graph, $G=(V,E)$, where $V$ is the set of vertices, and $E$ is the set of edges. The set $V$ is then partitioned into two sets, $V=X \cup Y$, corresponding to the rows $X$ and columns $Y$ of the data matrix $A$, and the weight of the $E$ edges corresponds to the elements $a_{ij}$ of $A$. As illustrated in Figure 31, biclustering can be used to discover maximal cliques from binary or real-valued matrices in this bipartite graph (bicliques) [1]. While less popular than the matricial view, the graph view is used for algorithm development [56, 112, 235], and to visually interpret biclustering solutions [158].

### Criteria to select a biclustering algorithm

The rapid development of biclustering algorithms implies that it is not feasible to test every available algorithm when applying and evaluating biclustering. The choice of an adequate biclustering algorithmis crucial, as the characteristics of the biclustering solution depend on the algorithm’s properties. In this section, we discuss seven aspects to consider when selecting a biclustering algorithm for analysis: *data domains*, *data input*, *computational efficiency*, *dimensional bias*, *robustness to distortions*, *the positioning of biclusters*, and *data-driven aspects*.

The **data domains** are a significant consideration when selecting an appropriate algorithm [7], as the algorithms are not equally tuned to detect patterns in different data domains. An algorithm developed and tested for a specific data domain (such as analyzing transcriptional profiles [236] or protein–protein interaction networks [237]) can theoretically be used for other tasks. However, this does not mean that it is equally suited for distinct application domains due to the differences in terms of data homogeneity [7]. Algorithms optimized to detect constant biclusters [12] may be unsuited for coherent [89] or order preserved [60] biclusters. Additionally, analyzing weighted biological networks implies different algorithms than unweighted networks [9, 39]. Comparative studies help bridge the gap between algorithm development and application studies by evaluating the algorithms on both synthetic data with well-defined coherences and real-world datasets, often considering measures of biological significance [3, 20, 23–27].

Associated with the previous topic is the **data input** of each algorithm. Biclustering algorithms were traditionally designed to operate on *real-valued matrices*. With the growth of biclustering research, there are now algorithms prepared to detect patterns on *discrete* [8, 58], *binary* [64, 65], *categorical* [8, 46], and *heterogeneous* [11, 41] datasets. This implies that particular care must be considered when *(1)* selecting the preprocessing techniques to apply to the data for each algorithm under use, and *(2)* selecting an adequate algorithm considering the format of the data matrix.

The **computational efficiency** of each algorithm is another aspect to ensure a reasonable consumption of computational resources. Studies should evaluate the computational cost in terms of computational complexity [18, 21, 22], and running time [3, 20, 24, 25].

Closely associated with computational efficiency is the strategy to handle **dimensional bias** [238]. This bias implies that using the biclustering algorithms on either the original data matrix or its transpose is not irrelevant, which has two consequences. The first consequence is related to *optimization*: the use of resources will depend on the shape of the matrix, which must be considered when applying the biclustering algorithm in either the original or transposed matrix [182]. Second, an algorithm’s *coherency* depends on the data matrix’s orientation, which should be considered [3, 8]. For example, an algorithm additive on rows can be additive on columns if the transposed matrix is considered, implying adequate data preprocessing.

Depending on the characteristics of the data to be mined, the **robustness to distortions** of each algorithm must be considered. These distortions are associated with *missing values*, with different algorithms having different strategies (or no strategy) to handle then [20], and to detect homogeneous structures despite the presence of *noise* in the datasets [3, 20, 24, 25].

The **positioning** of the biclustering solution is a critical aspect when selecting the biclustering algorithm, as not all algorithms guarantee coverage of all rows and columns in the matrix [21, 22]. Additionally, overlapping between biclusters implies additional challenges for biclustering interpretation [158].

Finally, **data-driven aspects** influence the choice of an adequate algorithm. For example, for the mining of temporally contiguous biclusters in time series datasets ($Observations \times Time Points$), specific algorithms must be considered to mine the temporal patterns [11, 73, 74, 239]. Another example is incorporating constraints derived from domain-based knowledge to improve the performance of the algorithms [6].

### Interpretability of a biclustering solution

A biclustering algorithm applied to a data matrix returns a biclustering solution. In this section, we discuss challenges to guarantee that the biclusters are meaningful. Biclustering algorithms face the risk of both **overfitting** and **underfitting**. This risk is highly associated with the number and size of the obtained biclusters. For example, while FABIA discovers several biclusters that must be smaller than the number of rows of the dataset, BicPAM discovers several biclusters that can be orders of magnitude higher than the number of rows or columns of the data matrix [59, 89].

As different algorithms discover a highly different number of biclusters, biclustering analysis is prone to under and overfitting. An algorithm that discovers a reduced number of large biclusters is prone to underfitting, while an algorithm that discovers many small biclusters is at risk of overfitting. Table 12 explains strategies for overcoming underfitting and overfitting in biclustering analysis.

**Table 12:** Underfitting and overfitting are two issues for biclustering analysis; this table illustrates possible strategies to both identify and overcome these issues

	Identifying it	Solving strategies
Underfitting	The algorithm discovered a small number of large biclusters; The biclusters are not statistically significant; The biclusters do not have a clear pattern (high noise).	Remove uninteresting rows or columns; Changing the parametrization of the algorithm to be more restrictive; Use a biclustering algorithm that parametrizes the size of the biclusters; Apply biclustering a second time in each sub-matrix; Manually crop the biclusters.
Overfitting	The algorithm discovered a large number of small biclusters; The biclusters are not statistically significant.	Preprocess the data matrix to have discretized values; Configure the algorithm to obtain less algorithms; Use an algorithm that is less prone to overfitting; Filter out uninteresting biclusters; Group similar biclusters (meta-biclustering).

Given a biclustering solution, extracting actionable insights implies using adequate qualitative and quantitative measures, which are summarized in Table 13.

**Table 13:** Comparison between categories of evaluation measures

Visualization techniques	
Advantages	Give visual insights of the information inside the biclustering solution;
Disadvantages	It is a challenge to visualize a high number of (potentially overlapping) biclusters;
	They do not quantify the value of the bicluster;
**External Metrics**	
Advantages	Compare the similarity of the obtained solution with a reference;
Disadvantages	Require a reference solution;
**Internal Metrics**	
Advantages	Evaluate the internal homogeneity of the bicluster;
Disadvantages	Require to assume the coherency of the bicluster;
	Small biclusters can have high homogeneity by chance;
**Statistical Significance Metrics**	
Advantages	Evalute how relevant is the biclustering solution against a noisy background;
Disadvantages	Require assumptions of internal coherency
**Problem Specific Evaluation**	
Advantages	Allows the use of domain-specific techniques to evaluate results from biclustering analysis;
Disadvantages	Requires the integration of biclustering results into analysis pipelines, which is not a trivial task.

### Application domains

**Figure 32:**
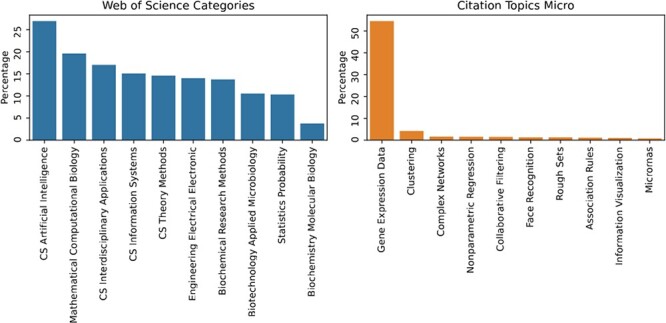
Main areas of biclustering research, showing the relevance of bioinformatics, and in particular, gene expression data; these charts consider results from the Web of Science database, when using *biclustering* and *coclustering* as keywords and limiting the search to 2000 (publication of the Cheng and Church algorithm).

Biclustering identifies subsets of rows and columns in a data matrix. This section explores the application of biclustering techniques in data analysis. As illustrated in Figure 32, bioinformatics, particularly the task of gene expression data analysis, is the primary application domain of biclustering. Beyond bioinformatics, biclustering is also widely used in other fields such as recommendation systems, text mining, and resource utilization. Table 14 illustrates general application areas of biclustering. Additionally, Table 15 highlights studies where biclustering is used to extract actionable insights in several applicational domains.

**Table 14:** The versatility of biclustering algorithms implies their use in several application domains

Domain	Biclustering is used to	References
Gene Expression	Functional annotate unclassified genes	[7, 11, 240]
	Identify disease subtypes	[7, 11, 241]
	Predict disease progression	[7, 11, 70]
Biological Networks	Identify sub-modules in homogeneous networks	[9, 242, 243]
	Identify sub-modules in heterogeneous networks	[9, 244, 245]
Physiological	Identify spatio-temporal patterns of brain activity and connectivity	[16, 26, 87]
	Group patients based on neurological variables	[246, 247]
Clinical	Identify clinical profiles based on disease progression	[75, 192, 193]
Bibliometric	Identify trends in academic literature	[248–250]
Text Mining	Identify groups on word that appear together in documents	[251–253]
Recommendation Systems	Recommend items based on user preferences	[13, 33]
Resources Utilization	Identify spatial-temporal patterns of consumption	[69, 88, 254]

**Table 15:** Data characteristics and research outcomes for biclustering in several data domains

Domain	Reference	Study objective	Biclustering Algorithm	Biclusters	Research Outcomes
Gene Expression	[241]	Identify sub-groups of patients	Plaid [99]	*Genes* $\times $ *Patients*	The biclustering solution was transformed into seven clusters of breast cancer patients based on their expression of immune-related genes.
	[269]	Understand the impact of a substance in the brain.	FABIA [59]	*Genes* $\times $ *Samples*	The biclusters show differences between control and experimental groups.
	[70, 71]	Classify response of patients to treatment.	CCC-Biclustering [11]	*Genes* $\times $ *Time*	Information biclusters improve the performance of classification problems.
Biological Networks	[66]	Predict protein-protein interactions.	Bimax [64]	*Protein* $\times $ *Protein*	The use of biclustering with rule mining extracted protein-proteins interactions not present in cancer databases.
	[9]	Identify biological modules	BicNET [234]	*Gene* $\times $ *Gene* and *Protein*$\times $*Protein*	The use of BicNET in biological modules discovered modules with heightened biological significance.
Agriculture	[265]	Evaluate differences between fermentation processes	double hierarchical clustering	*Treatments* $\times $ *VOCs*	The number of discovered biclusters are consistent with a biplot analysis.
	[53]	Identify biomarkers of organic farming	double hierarchical clustering	*DIESI-MS* $\times $ *Harvest*	Biclusters distinguish between organic and convencional farming.
Plant Biology	[54]	Do a codon usage bias analysis on rosales species.	double hierarchical clustering	*Plant Species* $\times $ *Genetic Codon*	Biclustering analysis identified three codons.
Physiological	[26]	Evaluate the capacity of biclustering when identifying brain regions interacting together over time.	Several	*Brain Region* $\times $ *Time*	BicPAM [89] and CCC-Biclustering [11] were found to be adequate algorithms.
	[16]	Build a genome-connectome bipartite graph model	N/A	*SNP* $\times $ *FNC*	Biclustering results suggest that somato-motor and visual brain areas provide insights into schizophrenia.
Clinical	[263]	Stratify patients	SUBSTRA [263]	*Patients* $\times $ *Transcripts*	The identified biclusters have phenotype-relevant patient subtypes.
	[270]	Identify disease progression patterns	BicPAM [89]	*Patients* $\times $ *clinical variables*	The biclusters improve the performance of classifiers at unraveling disease presentation patterns.
Bibliometric	[271]	Evaluate the treatment of sepsis by the literature	gCLUTO [272]	*MeSH terms* $\times $ *PMIDs*	Biclustering results show categories and several aspects for the treatment of sepsis.
	[250]	Evaluate postmenopausal osteoporosis research by the literature	gCLUTO [272]	*MeSH terms* $\times $ *PMIDs*	The etiology and drug treatment of postmenopausal osteoporosis are research hotspots.
Text Mining	[245]	Identify biological networks in research	EBC [273]	*Entity* $\times $ *Other Entity*	Results show broad themes for relationships between biological entities
	[253]	Detect the presence of clusters of rows in multiple documents.	PBBA [253]	*Words* $\times $ *Tweets*	Biclustering detected more realistic word connotations compared to clustering.
Recommendation Systems	[267]	Proposes a collaborative filtering approach.	Bimax and XMotifs [58, 64]	*users* $\times $ *items*	The incorporation of biclustering improves the performance of collaborative filtering process.
Resources Utilization	[69]	Identify water consumption patterns.	spectral biclustering, spectral co-clustering, e-CCC, LateBiclustering [73, 74, 111, 112]	*Sensor* $\times $ *Time*	Results show the effectiveness of biclustering detecting patterns of water consumption for strategic planning.
	[88]	Identify patterns of eletricity consumption	EBI [88]	*Sensor* $\times $ *Time*	Several electricity consumption patterns were discovered.
	[254]	Apply biclustering for mining traffic patterns of road mobility	BicPAM [89]	*Time* $\times $ *Places*	Biclustering algorithms successfully find statistically significant patterns of road mobility.

The most popular application of biclustering is the analysis of **gene expression** level according to several experimental conditions. In these data matrices, each row represents a gene, and its expression level is measured across a set of conditions (columns). Biclustering is then used to identify patterns only common in a subset of conditions or time points [1, 11]. Biclustering algorithms are applied to gene expression data for various tasks, such as functionally annotating unclassified genes [7, 240, 255], identifying disease subtypes [241, 256], and predicting disease prognostics in personalized medicine [192, 193, 257].

Another popular application domain of biclustering is the identification of sub-modules in **biological networks**. While biclustering cannot be directly applied to detect modules in graph structures, it can be applied whenever the data can be modeled as a weighted bipartite graph. As biological networks represent interactions between different biological entities (such as proteins, protein complexes, genes, metabolites, drugs, and diseases), biclustering identifies the interaction modules [9, 258]. In particular, biclustering is used to analyze both homogeneous and heterogeneous networks. Homogeneous networks refer to interactions between the same biological entities, such as protein–protein interaction [242, 243, 258–260], while heterogeneous networks measure interaction between distinct entities, such as functionally related genes/proteins [244] or genes/diseases [245].

In the analysis of **physiological data**, particularly in neurosciences, biclustering can be used to analyze signals from brain regions across a subset of stimuli responses over time [26, 87]. Furthermore, biclustering is used to analyze brain connectivity using extracted features from data [16, 19], and to group subjects based on the similarity of extracted features [246, 247].

A significant application of biclustering is the analysis of health records, which includes previously discussed gene expression and physiological data, as well as more generic **clinical Data**. Biclustering is used to stratify patients according to their similarity [75, 76]. Patients can have similar biological, demographic, or clinical attributes, which present opportunities to make medical and administrative decisions [71, 77, 261]. Biclustering was previously used to stratify patients based on their genomic profile [262, 263] and clinical profile [264], as well as to improve the performance of classifiers [71, 77].

The ability of biclustering to detect local modules suggests its use in **bibliometric analysis** to identify trends in academic literature. Using academic databases (such as PubMed) to extract studies, biclustering is then used to analyze the presence of common keywords in articles and identify trends in biomedical research [248–250].

Other areas of biclustering research in biological and biomedical domains include **epidemiologic research**, where it is used to identify trends in disease progression, either by analyzing the temporal evolution of diseases in specific regions [85] or by identifying spatial trends of disease [84], evaluating the effects of treatments in **precision agriculture** [52, 53, 265], and identifying biological patterns in plant biology [54, 266].

Beyond biological and biomedical domains, text mining, recommendation systems, and resource utilization are the most popular areas of biclustering research.

**Text mining** has a similar application as bibliometric analysis, as biclustering is applied to the numerically converted versions of text documents to identify groups of words that appear together over documents [251–253].

In **recommendation systems**, biclustering is used with one of two possible objectives: either recommend items that belong to the same bicluster as the user or recommend items similar to the ones in the bicluster [267]. For a survey on biclustering applied to recommendation systems, see either Singh [13] or Singh and Mehrotra [33].

Biclustering is also used to identify patterns of **resources utilization**. This includes the temporal analysis of a diversity of attributes such as water [69], energy consumption [88], or urban mobility patterns [68, 254, 268].

## Conclusion

Biclustering discovers local relationships hidden as sub-matrices in a data matrix, allowing the identification of complex patterns by recognizing that relationships between observations occur within subsets of attributes. This capability makes biclustering a powerful tool for uncovering intricate structures, including the simultaneous membership of observations in multiple biclusters and groups.

Biclustering can be applied whenever data have the form of a matrix, discovering subspaces of the original matrix that satisfy a criterion of homogeneity and statistical significance. The flexibility offered by biclustering made it a popular approach for pattern discovery and identification of modules in both descriptive and predictive learning tasks in bioinformatics.

The first contribution of this survey is a taxonomy of theoretical concepts for biclustering, divided into four categories: *bicluster*, *biclustering solution*, *biclustering algorithm*, and *evaluation measures*.

A *bicluster* is the atomic element of biclustering data analysis. Depending on the attributes constituting the bicluster, it is classified as homogeneous (numeric, categorical, and binary) or heterogeneous (reflecting attributes beyond the traditional homogeneous tabular data). In a bicluster, coherency reflects the correlation between the bicluster elements (with distinct biological interpretations). Additional aspects of a bicluster are its contiguity, which is relevant for domains such as time series analysis, and the bicluster pattern that simplifies its interpretation.

The *biclustering solution* is the set of biclusters obtained by a biclustering algorithm. The overlapping between biclusters is relevant for interpretability. While nonoverlapped solutions have a more straightforward interpretation (since their positioning is similar to traditional clustering), recent research trends focus on arbitrarily positioned biclusters since these algorithms outperform nonoverlapping algorithms in comparative analysis. Plaid models often formulate the overlapping between bicluster elements, reflecting the cumulative effect of biological processes.

A *biclustering algorithm* refers to the process that mines the biclustering solution. Biclustering has efficiency challenges due to the combinatorial nature of discovering subspaces. Therefore, developing efficient algorithms is crucial given the datasets’ current and expected growth in size and complexity. Since biclustering is unsupervised, the algorithms should be flexible (able to find an arbitrary number of biclusters), robust (capable of handling noise and missing values), and able to detect biclusters with guarantees of statistically significant.

Qualitative and quantitative measures should be considered in what concerns *evaluation measures*, or how to analyze, quantify, and interpret biclustering solutions. Recent biclustering algorithms can mine many biclusters, raising challenges for biclustering interpretation and visualization. It is also relevant to note that biclustering algorithms are often integrated within an analysis pipeline, and expecting them to be prepared for a hundredfold increase in the number of generated biclusters is not reasonable.

The second part of this survey is associated with the challenge of using biclustering in real-world applications and its integration into a data mining pipeline that should be efficient and effective in delivering actionable knowledge. We identify *application domains* where biclustering is prominent, particularly in bioinformatics, discuss *criteria to select an algorithm* when applying and evaluating biclustering, and relate biclustering with other data mining tasks (clustering, pattern mining, classification, triclustering, and N-way clustering).

**Key Points**

- Biclustering methods generalize traditional clustering by discovering local interactions between observations;
- Biclustering is state-of-the-art in biological and biomedical domains and is further used in text mining, recommendation systems, and spatiotemporal domains;
- We provide a comprehensive overview of biclustering and its main components (Bicluster, Biclustering Solution, Biclustering Algorithms, Evaluation Measures) and applications;
- We integrate contributions from several studies, together with new concepts in a unified taxonomy of biclustering applied to bioinformatics;
- We present applicational aspects of biclustering, prominent domains of analysis, a guide to selecting an adequate biclustering algorithm, and a relation with other data mining tasks.
